# Double Sampling for Informatively Missing Data in Electronic Health Record‐Based Comparative Effectiveness Research

**DOI:** 10.1002/sim.10298

**Published:** 2024-12-05

**Authors:** Alexander W. Levis, Rajarshi Mukherjee, Rui Wang, Heidi Fischer, Sebastien Haneuse

**Affiliations:** ^1^ Department of Statistics & Data Science Carnegie Mellon University Pittsburgh Pennsylvania; ^2^ Department of Biostatistics Harvard T. H. Chan School of Public Health Boston Massachusetts; ^3^ Department of Population Medicine Harvard Pilgrim Health Care Institute and Harvard Medical School Boston Massachusetts; ^4^ Department of Research and Evaluation Kaiser Permanente Pasadena California USA

**Keywords:** causal inference, double sampling, missing data, semiparametric theory, study design

## Abstract

Missing data arise in most applied settings and are ubiquitous in electronic health records (EHR). When data are missing not at random (MNAR) with respect to measured covariates, sensitivity analyses are often considered. These solutions, however, are often unsatisfying in that they are not guaranteed to yield actionable conclusions. Motivated by an EHR‐based study of long‐term outcomes following bariatric surgery, we consider the use of double sampling as a means to mitigate MNAR outcome data when the statistical goals are estimation and inference regarding causal effects. We describe assumptions that are sufficient for the identification of the joint distribution of confounders, treatment, and outcome under this design. Additionally, we derive efficient and robust estimators of the average causal treatment effect under a nonparametric model and under a model assuming outcomes were, in fact, initially missing at random (MAR). We compare these in simulations to an approach that adaptively estimates based on evidence of violation of the MAR assumption. Finally, we also show that the proposed double sampling design can be extended to handle arbitrary coarsening mechanisms, and derive nonparametric efficient estimators of any smooth full data functional.

## 1 Introduction

Missing data is a well‐studied problem, with researchers having a vast array of statistical methods at their disposal including inverse‐probability weighting (IPW) [1], multiple imputation [2], and doubly‐robust methods [3, 4]. For the majority of these, a missing at random (MAR) assumption [5] is, in one way or another, invoked. For settings where an MAR assumption is not viewed as plausible, methods exist based on alternative sets of identifying assumptions [6], availability of an instrumental variable [7] or a “shadow variable” [8], sensitivity analyses [9] and the estimation of bounds [10]. Interestingly, common to all of these methods is that they approach the task of dealing with missing data at the analysis stage, that is with an exclusive focus on methods for the data at‐hand.

An alternative strategy is to engage in additional data collection, referred to in this paper as *double‐sampling*, specifically to obtain information that could either inform the plausibility of missingness assumptions or be used in an analysis to mitigate bias, or both. Such a strategy is common when addressing confounding [11] and measurement error and/or missclassification [12, 13], but seems to have been under‐explored as a strategy for addressing missing data [14, 15, 16, 17, 18]. Moreover, a general treatment of double sampling for missing data in the context of causal inference has not been developed.

One important area of biomedical and public health research where missing data is almost ubiquitous is that of studies making use of electronic health records (EHR). With large sample sizes and rich covariate information over extended periods, EHR data represent a significant and cost‐effective opportunity [19]. Furthermore, these data present a key alternative when randomized clinical trials are not feasible or could not be conducted ethically. EHR systems, however, are typically designed to support clinical and/or billing activities, and not for any particular research agenda. As such, investigators who wish to use EHR data must deal with potential threats to validity including, as mentioned, missing data. Moreover, whether a particular data element is observed in an EHR is likely dependent on the complex interplay of numerous factors [20], which may, in turn, cast doubt on the plausibility of the MAR assumption. In such settings, augmentation of the EHR with additional information via double sampling may be especially helpful [21]. Indeed, Koffman et al. [22] recently reported on a telephone‐based survey used to obtain additional information for use in an investigation of the association between bariatric surgery and five‐year weight outcomes using data from an EHR [23]. Key to the latter was the fact that many subjects who had undergone bariatric surgery disenrolled from their health plan before their five‐year post‐surgery date. Towards understanding the reasons for disenrollment and to evaluate the MAR assumption, the investigators conducted the telephone‐based survey to obtain the otherwise missing weight information and other relevant factors. Although their report focuses on disenrollment in relation to missingness, the authors did stress the potential for using the augmented data to correct an otherwise invalid analysis (i.e., of the association between bariatric surgery and weight at follow‐up), but identified the need for novel statistical methods to be developed.

Motivated by this backdrop, we consider double sampling as a means to deal with potentially informatively missing or MNAR data. Specifically, we present novel identification results for the causal average treatment effects in observational settings with missing outcome data. Based on these we describe a suite of five analysis strategies for the context we consider, each distinguished by the nature of the data that is taken to be available, the assumptions that analysts are required to make and the estimator that is to be employed. For the proposed strategies that, to‐date, have not been formally described, we establish asymptotic results and characterize efficiency and robustness properties. Finally, we generalize many of these results to allow for arbitrary coarsening of the desired complete data of interest, where complete data are recovered on a subsample via intensive follow‐up. Note, throughout, when not provided in the text, detailed proofs are presented in Appendices A and B in the Supporting Information.

## 2 A Hypothetical EHR‐Based Study

### 2.1 Context, Notation, and Terminology

To anchor the methods we propose, consider a hypothetical EHR‐based study for which the goal is to compare two bariatric surgery procedures (e.g., Roux‐en‐Y gastric bypass vs. sleeve gastrectomy) in relation to three‐year weight change outcomes. To that end, we assume that appropriate inclusion/exclusion criteria have been specified and operationalized to identify all patients in the EHR who are ‘eligible’ for the study, resulting in a sample of size n which is taken to be a random sample from the population of interest.

Formally, let A∈𝒜, with |𝒜|<∞, denote the treatment and Y∈ℝ the outcome of interest. In the hypothetical study, A represents the type of surgery, and Y the change in BMI at three years post‐surgery (slightly different from the study described above [22]) relative to baseline. Then, let Y(a) denote the potential outcome or counterfactual had we fixed treatment level A=a, for a∈𝒜. We take the target parameter of interest to be some contrast among the mean counterfactuals, 𝔼[Y(a)]. In the hypothetical bariatric surgery study, for example, a natural contrast would be the average treatment effect (ATE), 𝔼[Y(1)]−𝔼[Y(0)]. Throughout this work, towards estimating 𝔼[Y(a)] using the data from the EHR, we invoke the usual ‘causal’ identifying assumptions of consistency, no unmeasured confounding and positivity [24]. Regarding the control of confounding bias, we assume that a sufficient set of confounders $L\in {\mathbb{R}}^{d}$, available in the EHR, has been identified to render the assumption of no unmeasured confounding plausible.

For the setting just described, we refer to (L,A,Y) as the *complete data* and conceive it as arising from some joint distribution, $P_{c}$. Given an i.i.d sample of size n from $P_{c}$ one could estimate 𝔼[Y(a)] by, say, targeting the g‐formula functional, $\chi_{a}(P_{c})\;=\;{𝔼}_{P_{c}}[{𝔼}_{P_{c}}[Y|L,A=a]]$, which identifies 𝔼[Y(a)] under the aforementioned causal assumptions [25]. For example, one could use the plugin estimator to estimate the ATE via $\hat{\text{ATE}}\;=\;\chi_{1}(\hat{P_{c}})-\chi_{0}(\hat{P_{c}})$, relying on the fit of an outcome regression model for ${𝔼}_{P_{c}}[Y|L,A=a]$.

To complete the context we consider, we assume that, while L and A are measured on all patients, the outcome is only partially observed; that is for some patients the value of Y is missing. In the hypothetical example this may arise because a patient disenrolled from the health plan prior to the 3‐year post‐surgery date or because they did not have an encounter within some (reasonable) window of the date. Formally, let R∈{0,1} be an indicator for the observance of Y in the EHR; at the outset, therefore, the information that is readily available consists of n i.i.d replicates of (L,A,R,RY), referred to as the *incomplete data*.

### 2.2 Analysis Strategy #1

Given incomplete data, one way forward is to combine a complete data strategy, which one would use had such data been available, with some approach for ‘dealing’ with the missing data. For example, one could combine the use of the g‐formula indicated above with either inverse‐probability weighting based on a model for R or multiple imputation for the missing values of Y. In addition to the usual causal assumptions, the validity of such a procedure will hinge on a MAR assumption, such as:

Assumption 1 (missing at random outcomes) R⫫Y|L,A.

Crucially, with this assumption in hand, one can proceed as would be done otherwise on the basis of those individuals with R=1, since the distribution of Y|L,A is the same as Y|L,A,R=1—that no information on the distribution of Y|L,A,R=0 is available can be safely ignored. For instance, one can employ the g‐formula on the basis of the complete‐case outcome model 𝔼[Y|L,A=a,R=1].

### 2.3 The Potential for MNAR

Suppose, however, that a discussion among the collaborators at the design stage of the study (i.e., at the time the study is being planned and/or a grant/proposal is being written) raises the possibility that the outcome data are MNAR; that is, that Assumption 1 may not hold with respect to the baseline covariates L that will be available. In the hypothetical bariatric surgery study, for example, it may be that patients with worse outcomes (in a manner beyond what can be predicted with L and A) interact more often with the health care system, and thus have less missing data and/or are less likely to disenroll from their health plan. It is also possible that subjects with worse outcomes are more likely to drop out, perhaps to receive care outside of their original health plan.

The key challenge that a violation of Assumption 1 poses is that the distribution of Y|L,A,R=0 can no longer be safely ignored, and yet there is no information to learn about it. Since MNAR is not testable, the literature on methods for data that are MNAR has generally focused on frameworks for sensitivity analyses and analyses that directly provide bounds on the effect of interest. An alternative to these typically post‐hoc approaches, especially if the potential for MNAR is established early in the research process, is to engage in additional data collection efforts that are specifically and preemptively tailored to being able (at least partially) to move ‘beyond’ MNAR.

## 3 Double Sampling When MNAR is Suspected

Central to the proposed work is that follow‐up is performed for a subsample of the patients for whom R=0, and that the corresponding (otherwise missing) value of Y is ascertained. While such additional data collection is employed in a wide range of settings, in this paper we follow Frangakis and Rubin [15] and use the label *double sampling*.

### 3.1 Notation and Terminology

**FIGURE 1:**
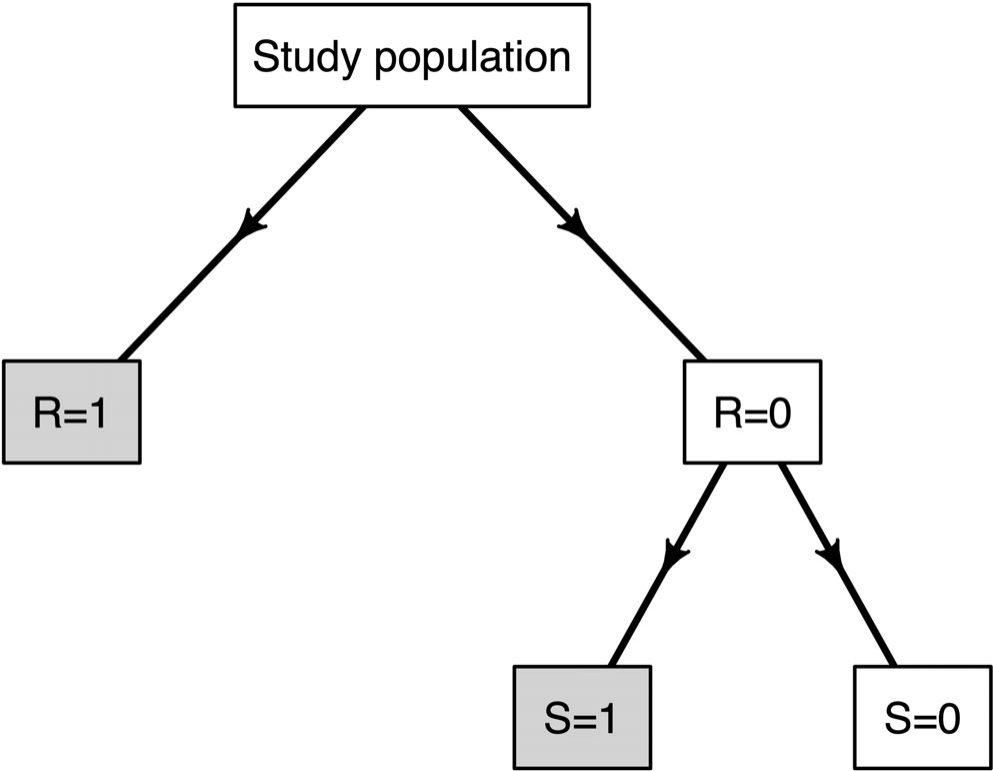
Schematic illustrating the double sampling study design. Gray boxes indicate subgroups for which the outcome is observed: either subjects are observed in the EHR directly (R=1) or are selected in the follow‐up sample (R=0 and S=1).

Let S∈{0,1} be an indicator for whether a given patient is selected into the follow‐up subsample and outcome data are successfully obtained. Note, by design and as can be seen from Figure 1, S≡S(1−R); S can only be 1 if R=0 and is equal to 0 deterministically if R=1. With this notation, we refer to O=(L,A,R,S,(R+S)Y) as the *final observed data* and the corresponding joint distribution by $P_{o}$. Throughout this section, we assume that we observe a random sample $O_{1},\ldots ,O_{n}\overset{\text{iid}}{∼}P_{o}$.

To complete terminology, we refer to (L,A,Y,R,S) as the *full data* and denote the corresponding joint distribution by $P_{f}$. Note, both $P_{c}$ and $P_{o}$ are induced by $P_{f}$ via appropriate marginalization. As will become clear, the use of distinct labels is to help clarify where and how key identifying conditions are employed.

### 3.2 Identification

As with analysis strategy #1, we proceed by specifying assumptions that permit the identification of the complete data distribution, $P_{c}$, despite only having access to the final observed data. Specifically, consider the following assumptions:

Assumption 2 (no informative second‐stage selection) S⊥⊥Y|L,A,R=0.

Assumption 3 (positivity of second‐stage selection probabilities) For some ϵ>0, it holds that $P_{o}[S=1|L,A,R=0]\geq \epsilon ,P_{o}$‐almost surely.

Note, Assumption 2 can be viewed as an MAR‐type assumption, specifically in relation to the mechanism underpinning who is selected to be double‐sampled and successfully followed‐up, and, thus, represents an alternative to or replacement for the usual MAR assumption regarding R.

Based on these, consider the following identification result:

Proposition 1 *Under Assumptions* *2* *and* *3*, *the full data distribution* $P_{f}$ *is identified from the final observed data distribution* $P_{o}$.

Let $p_{o}=\partial P_{o}/\partial \mu$ and $p_{f}=\partial P_{f}/\partial \mu$ denote the densities for $P_{o}$ and $P_{f}$, respectively, both with respect to some dominating measure μ. We can then factor the full data distribution as:

$$\begin{array}{ll} & p_{f}(L,A,R,S,Y)\\ & \;=\;p_{f}(L,A,R,S,RY,(1-R)Y)\\ & \;=\;p_{o}(L,A,R,S,RY)\;p_{f}{\left(Y|L,A,R=0,S\right)}^{1-R}\\ & \;=\;p_{o}(L,A,R,S,RY)\;p_{o}{\left(Y|L,A,R=0,S=1\right)}^{1-R} \end{array}$$

with the last of these steps enabled by Assumption 2. Since the last expression depends solely on $p_{o}$, it follows that $P_{f}$ is identified by $P_{o}$. Note, we may safely introduce S=1 in the conditioning event by Assumption 3.

An immediate consequence of Proposition 1 is that the complete data distribution, $P_{c}$, is identified and, hence, any functional depending on $P_{c}$ is identified. Therefore, given Assumptions 2 and 3, one can use the final observed data to estimate quantities of interest, such as mean counterfactuals. This observation, in turn, is the basis for a series of additional analysis strategies proposed in the remainder of this section.

### 3.3 Analysis Strategy #2

Given an i.i.d sample of O=(L,A,R,S,(R+S)Y), Proposition 1 implies that, if Assumptions 2 and 3 hold, we can nonparametrically identify 𝔼[Y(a)] via the g‐formula representation:

(1) $$\tau_{a}(P_{o})={𝔼}_{P_{o}}[\mu_{a,R}(L)\gamma_{a}(L)+\mu_{a,S}(L)(1-\gamma_{a}(L))]$$

with $\gamma_{a}(L)=P_{o}(R=1|L,A=a)$, $\mu_{a,R}(L)={𝔼}_{P_{o}}[Y|L,A=a,R=1]$, and $\mu_{a,S}(L)={𝔼}_{P_{o}}[Y|L,A=a,S=1]$. With this representation, one can construct an estimator of the ATE by targeting $\tau_{a}(P_{o})$. One simple approach would be to estimate each component nuisance function, that is $\gamma_{a}(L)$, $\mu_{a,R}(L)$ and $\mu_{a,S}(L)$, via parametric modeling and combine via an empirical version of expression (1). In the following, we derive and characterize a nonparametric efficient and multiply robust estimator ${\hat{\tau }}_{a}$ of $\tau (P_{o})$. As we will see, efficient estimation additionally requires a model for the treatment probability $\pi_{a}(L)=P_{o}(A=1|L)$, as well as for the double sampling probabilities $\eta_{a,0}(L)=P_{o}(S=1|L,A=a,R=0)$. Note, as we will prove, this approach has the advantage that under relatively mild conditions, $\sqrt{n}$‐rate convergence can still be attained while using flexible machine learning‐based models for each nuisance function. The following result is a straightforward application of Proposition A.2 proved in Appendix A of the Supporting Information.

Theorem 1 *Let* $\mu_{a}(L)=\mu_{a,R}(L)\gamma_{a}(L)+\mu_{a,S}(L)(1-\gamma_{a}(L))$. *The nonparametric influence function with respect to the maximal tangent space of* $\tau_{a}(P_{o})$ *is*

$$\begin{array}{ll} & {\dot{\tau }}_{a}(O;P_{o})\\ & \;=\mu_{a}(L)-\tau_{a}(P_{o})+\frac{1(A=a)}{\pi_{a}(L)}\left\{\left(R+\frac{S}{\eta_{a,0}(L)}\right)Y-\mu_{a}(L)\right.\\ & \;\left.+(1-R)\left(1-\frac{S}{\eta_{a,0}(L)}\right)\mu_{a,S}(L)\right\} \end{array}$$

With this efficient influence function in hand, one can proceed by using the standard one‐step estimator [26, 27], specifically:

$${\hat{\tau }}_{a}=\tau_{a}({\hat{P}}_{o})+\frac{1}{n}\overset{n}{\underset{i=1}{\sum }}{\dot{\tau }}_{a}(O;{\hat{P}}_{o})$$

For simplicity we assume in the following results that the nuisance functions in ${\hat{P}}_{o}$ are trained on a separate independent sample. In practice, one can use cross‐fitting which involves splitting the data into training and test folds, fitting ${\hat{P}}_{o}$ on the training fold, and computing the one‐step estimator in the test fold [27, 28]. Full efficiency can be recovered by swapping the roles of the folds and averaging the resulting estimators [29]. The following result is obtained directly from Theorem A.1 and Proposition A.4 (see Appendix A of the Supporting Information):

Theorem 2 *Suppose* $\left\|{\dot{\tau }}_{a}(\;\cdot \;;{\hat{P}}_{o})-{\dot{\tau }}_{a}(\;\cdot \;;P_{o})\right\|=o_{P}(1)$. *Then*

$$\begin{array}{l} {\hat{\tau }}_{a}-\tau_{a}(P_{o})=O_{P}\left(\frac{1}{\sqrt{n}}+{\text{Bias}}_{\tau_{a}}({\hat{P}}_{o};P_{o})\right), \end{array}$$

*where*

$$\begin{array}{ll} & {\text{Bias}}_{\tau_{a}}({\tilde{P}}_{o};P_{o})\\ & ={𝔼}_{P_{o}}\left(\left(1-\frac{\pi_{a}(L)}{{\tilde{\pi }}_{a}(L)}\right)\left({\tilde{\mu }}_{a}(L)-\mu_{a}(L)\right)\right)\\ & \;+{𝔼}_{P_{o}}\left((1-\gamma_{a}(L))\frac{\pi_{a}(L)}{{\tilde{\pi }}_{a}(L)}\left(1-\frac{\eta_{a,0}(L)}{{\tilde{\eta }}_{a,0}(L)}\right)\left({\tilde{\mu }}_{a,S}(L)-\mu_{a,S}(L)\right)\right). \end{array}$$

*for any (fixed)* ${\tilde{P}}_{o}$. *Moreover, if* ${\text{Bias}}_{\tau_{a}}({\hat{P}}_{o};P_{o})=o_{P}(n^{-1/2})$, *then* $\sqrt{n}({\hat{\tau }}_{a}-\tau_{a}(P_{o}))\overset{d}{\to }𝒩(0,V)$, *where* $V={\mathrm{Var}}_{P_{o}}({\dot{\tau }}_{a}(O;P_{o}))$ *is the nonparametric efficiency bound*.

In addition to consistency, asymptotic normality, and efficiency, Theorem 2 reveals a set of robustness properties of ${\hat{\tau }}_{a}$. Specifically, observe that ${\text{Bias}}_{\tau_{a}}({\tilde{P}}_{o};P_{o})=0$ if: (i) $\left({\tilde{\mu }}_{a,R},{\tilde{\mu }}_{a,S},{\tilde{\gamma }}_{a})=(\mu_{a,R},\mu_{a,S},\gamma_{a}\right)$; (ii) $\left({\tilde{\mu }}_{a,S},{\tilde{\pi }}_{a})=(\mu_{a,S},\pi_{a}\right)$; or (iii) $\left({\tilde{\pi }}_{a},{\tilde{\eta }}_{a,0})=(\pi_{a},\eta_{a,0}\right)$. In particular, if the double sampling probabilities $\eta_{a,0}$ are known by design, then ${\text{Bias}}_{\tau_{a}}({\tilde{P}}_{o};P_{o})=0$ if $\left({\tilde{\mu }}_{a,R},{\tilde{\mu }}_{a,S},{\tilde{\gamma }}_{a})=(\mu_{a,R},\mu_{a,S},\gamma_{a}\right)$ or ${\tilde{\pi }}_{a}=\pi_{a}$. This robustness extends to the rate of convergence of the estimator (as in Rotnitzky, Smucler, and Robins [30]), in that the asymptotic bias term is bounded under mild conditions by a sum of product of $L_{2}(P_{o})$‐errors in nuisance function estimation: $\left\|{\hat{\pi }}_{a}-\pi_{a}\|\cdot \|{\hat{\mu }}_{a}-\mu_{a}\right\|$ + $\left\|{\hat{\eta }}_{a,0}-\eta_{a,0}\|\cdot \|{\hat{\mu }}_{a,S}-\mu_{a,S}\right\|$, where $\left\|f{\|}^{2}={𝔼}_{P_{o}}(f{\left(O\right)}^{2}\right)$ for any function f. In particular, if ${\hat{\pi }}_{a}$, ${\hat{\mu }}_{a}$, ${\hat{\eta }}_{a,0}$, and ${\hat{\mu }}_{a,S}$ are each $L_{2}(P_{o})$‐consistent at rate at least $n^{-1/4}$, then (under mild conditions) ${\hat{\tau }}_{a}$ is $\sqrt{n}$‐consistent and asymptotically efficient.

Another appealing consequence of the asymptotic normality result in Theorem 2 is that simple Wald‐type asymptotically valid confidence intervals are immediately available. For example, we can estimate $\mathrm{Var}({\hat{\tau }}_{a})$ with $\hat{\mathrm{Var}}({\hat{\tau }}_{a})=\frac{1}{n}\hat{V}=\frac{1}{n^{2}}\sum_{i=1}^{n}{\left({\dot{\tau }}_{a}(O_{i};{\hat{P}}_{o})\right)}^{2}$, with corresponding Wald‐type confidence interval given by ${\hat{\tau }}_{a}\pm z_{1-\alpha /2}\sqrt{\hat{\mathrm{Var}}({\hat{\tau }}_{a})}$, where $z_{\alpha }$ denotes the α‐quantile of the standard normal distribution.

### 3.4 Analysis Strategy #3

A key feature of ${\hat{\tau }}_{a}$ is that there was no need to invoke the usual MAR assumption for R; indeed, no assumptions regarding R are invoked. Suppose, however, that following data collection via double‐sampling, the investigative team decides that MAR Assumption 1 may indeed be plausible. It may be, for example, that new information regarding the mechanisms that underpin R becomes available; see Section 4. Alternatively, suppose the MAR assumption was always viewed as being potentially plausible and that the additional data was collected for the purpose of gaining efficiency in estimating the parameter of interest. In either of these settings, combining Assumption 1 with S≡S(1−R) and Assumption 2, gives that (R,S)⊥⊥Y|L,A. With this, one can nonparametrically identify 𝔼[Y(a)] via the g‐formula representation:

$$\tau_{a}^{*}(P_{o})={𝔼}_{P_{o}}[\mu_{a,\text{MAR}}(L)],$$

where $\mu_{a,\text{MAR}}(L)={𝔼}_{P_{o}}[Y|L,A,R+S=1]$. As in Section 3.4, while we could construct an estimator of $\tau_{a}^{*}(P_{o})$ based on a parametric model for the nuisance function $\mu_{a,\text{MAR}}(L)$, we derive a semiparametric efficient estimator of $\tau_{a}^{*}(P_{o})$ that is of a robust augmented IPW form. The following result is proved in Appendix B of the Supporting Information.

Theorem 3 *Under Assumptions* *1* *and* *2*, *and with* S≡S(1−R), *the semiparametric efficient influence function of* $\tau_{a}^{*}(P_{o})$ *is*

$$\begin{array}{ll} & {\dot{\tau }}_{a}^{*}(O;P_{o})\\ & \;=\mu_{a,\text{MAR}}(L)-\tau_{a}^{*}(P_{o})+\frac{1(A=a)(R+S)}{\pi_{a}(L)\{\gamma_{a}(L)+(1-\gamma_{a}(L))\eta_{a,0}(L)\}}\\ & \;\times (Y-\mu_{a,\text{MAR}}(L)) \end{array}$$

We remark that the influence function in Theorem 3 generalizes that discussed in Gilbert, Yu, and Rotnitzky [31], where they pursue two‐phase sampling for outcomes that are expensive to collect—in particular, they assume $P_{o}[S=0]=1$ and $\pi_{a}$ is fixed and known. With the result of Theorem 3, one can again proceed using the standard one‐step estimator, specifically:

$${\hat{\tau }}_{a}^{*}=\tau_{a}^{*}({\hat{P}}_{o})+\frac{1}{n}\overset{n}{\underset{i=1}{\sum }}{\dot{\tau }}_{a}^{*}(O;{\hat{P}}_{o})$$

the asymptotic properties of which are established in the following theorem, itself a straightforward consequence of Theorem A.1 and Proposition A.4 in Appendix A of the Supporting Information.

Theorem 4 *Suppose* $\left\|{\dot{\tau }}_{a}^{*}(\;\cdot \;;{\hat{P}}_{o})-{\dot{\tau }}_{a}^{*}(\;\cdot \;;P_{o})\right\|=o_{P}(1)$. *Then*

$${\hat{\tau }}_{a}^{*}-\tau_{a}^{*}(P_{o})=O_{P}\left(\frac{1}{\sqrt{n}}+{\text{Bias}}_{\tau_{a}^{*}}({\hat{P}}_{o};P_{o})\right),$$

*where*

$$\begin{array}{ll} & {\text{Bias}}_{\tau_{a}^{*}}({\tilde{P}}_{o};P_{o})\\ & \;={𝔼}_{P_{o}}\left[\left(1-\frac{\pi_{a}(L)\{\gamma_{a}(L)+(1-\gamma_{a}(L))\eta_{a,0}(L)\}}{{\tilde{\pi }}_{a}(L)\{{\tilde{\gamma }}_{a}(L)+(1-{\tilde{\gamma }}_{a}(L)){\tilde{\eta }}_{a,0}(L)\}}\right)\right.\\ & \;\times \left.\left({\tilde{\mu }}_{a,\text{MAR}}(L)-\mu_{a,\text{MAR}}(L)\right)\right] \end{array}$$

*for any* ${\tilde{P}}_{o}$. *Furthermore, if* ${\text{Bias}}_{\tau_{a}^{*}}({\hat{P}}_{o};P_{o})=o_{P}(n^{-1/2})$, *then* $\sqrt{n}({\hat{\tau }}_{a}^{*}-\tau_{a}^{*}(P_{o}))\overset{d}{\to }𝒩(0,V^{*})$, *where* $V^{*}={\mathrm{Var}}_{P_{o}}({\dot{\tau }}_{a}^{*}(O;P_{o}))$ *is the semiparametric efficiency bound under the assumptions of Theorem* *3*.

Note that analogous comments to those following Theorem 2 for ${\hat{\tau }}_{a}$ can be made here for ${\hat{\tau }}_{a}^{*}$ as well. First, robustness of ${\hat{\tau }}_{a}^{*}$ follows from the product bias elucidated in Theorem 4: ${\text{Bias}}_{\tau_{a}^{*}}({\tilde{P}}_{o};P_{o})=0$ if (i) ${\tilde{\mu }}_{a,\text{MAR}}=\mu_{a,\text{MAR}}$; or (ii) $\left({\tilde{\pi }}_{a},{\tilde{\gamma }}_{a},{\tilde{\eta }}_{a,0})=(\pi_{a},\gamma_{a},\eta_{a,0}\right)$. Moreover, ${\hat{\tau }}_{a}^{*}$ is $\sqrt{n}$‐consistent and asymptotically efficient if each nuisance function estimate is $L_{2}(P_{o})$‐consistent at rate at least $n^{-1/4}$. Second, we can estimate $\mathrm{Var}({\hat{\tau }}_{a}^{*})$ with $\hat{\mathrm{Var}}({\hat{\tau }}_{a}^{*})=\frac{1}{n}{\hat{V}}^{*}=\frac{1}{n^{2}}\sum_{i=1}^{n}{\left({\dot{\tau }}_{a}^{*}(O_{i};{\hat{P}}_{o})\right)}^{2}$, and construct the corresponding Wald‐type confidence interval.

### 3.5 Analysis Strategy #4

The key distinction between analysis strategies #2 and #3 is in relation to whether the MAR Assumption 1 holds. In practice there may not be a consensus as to whether it plausibly holds. For example, one collaborator may believe firmly that the outcomes were initially MAR given (L,A) while another may believe that there remains residual dependence of missingness status on the outcome (either directly or through some other, as‐yet unmeasured, factor). In this setting, one option may be to conduct a hypothesis test using the observed data to assess Assumption 1; while this assumption is untestable if all one has access to is the initially observed data, under Assumptions 2 and 3 the complete data distribution is identified and, in principle, MAR can be tested. Depending on the results of this test, one could report an analysis based on ${\hat{\tau }}_{a}$ which does not rely on Assumption 1, or ${\hat{\tau }}_{a}^{*}$ which does. While appealing in its simplicity, naïve use of the corresponding standard error estimator for the chosen estimator would not account for the uncertainty in the estimator selection. As such, inference will, in general, not be valid. Because of this, we do not derive any theory for this approach but do consider it as a comparator in the simulation study of Section 5.1.

### 3.6 Analysis Strategy #5

Finally, building on analysis strategy #4, one could proceed with a more explicitly data‐adaptive approach that selects between the two candidate estimators and provides valid post‐selection confidence intervals. To this end, we employ the recently developed methods of Rothenhäusler [32]. The approach from this work assumes that one has access to a collection of estimators, and that one ‘base’ estimator in this collection is consistent for the true target parameter of interest. At a high level, the methodology then anchors the analysis on this ‘base’ estimator, and chooses among the larger collection of estimators (some of which being potentially biased or highly variable) by minimizing a proxy for mean‐squared error.

Briefly, as an overview of the method in the one‐parameter case, consider estimation of a generic parameter $\theta_{0}(P)$ when there are k+1 asymptotically linear estimators ${\hat{\theta }}_{0},{\hat{\theta }}_{1},\ldots ,{\hat{\theta }}_{k}$, such that: (i) the ‘base’ estimator ${\hat{\theta }}_{0}$ is $\sqrt{n}$‐consistent for $\theta_{0}(P)$; and (ii) ${\hat{\theta }}_{j}$ is $\sqrt{n}$‐consistent for $\theta_{j}(P)$, for j=1,…,k, where $\theta_{j}(P)$ may or may not equal $\theta_{0}(P)$. With this collection of k+1 candidate estimators in hand, Rothenhäusler [32] proposed an estimator that selects among them by minimizing an estimate of the mean squared error. Formally, let ${\hat{\sigma }}_{j}^{2}$ be an estimator of the asymptotic variance of $\sqrt{n}({\hat{\theta }}_{j}-\theta_{j}(P))$, and ${\hat{\rho }}_{j}^{2}$ an estimator of the asymptotic variance of $\sqrt{n}({\hat{\theta }}_{j}-\theta_{j}(P)-{\hat{\theta }}_{0}+\theta_{0}(P))$. Then the procedure selects the ${\bar{j}}^{th}$ candidate estimator, where $\bar{j}=\underset{j}{\arg\;\min}\;\bar{R}(j)$ with $\bar{R}(j)=\max\{0,{\left({\hat{\theta }}_{j}-{\hat{\theta }}_{0}\right)}^{2}-{\hat{\rho }}_{j}^{2}/n\}+{\hat{\sigma }}_{j}^{2}/n$. Importantly, Rothenhäusler [32] derive asymptotically valid confidence intervals that take into account the uncertainty due to the selection procedure (see their Theorem 4).

Toward applying the Rothenhäusler [32] approach to the context of this paper, we take ${\hat{\tau }}_{a}$ of analysis strategy #2 to be the ‘base’ estimator (since it does not rely on Assumption 1), with ${\hat{\tau }}_{a}^{*}$ as an alternative estimator that is, in principle, more efficient than ${\hat{\tau }}_{a}$ if Assumption 1 does hold. Then, let $\hat{V}$ = $n\hat{\mathrm{Var}}[{\hat{\tau }}_{a}]$ and ${\hat{V}}^{*}$ = $n\hat{\mathrm{Var}}[{\hat{\tau }}_{a}^{*}]$, and define $\hat{Q}=\frac{1}{n}\sum_{i=1}^{n}{\left\{{\dot{\tau }}_{a}(O_{i};{\hat{P}}_{o})-{\dot{\tau }}_{a}^{*}(O_{i};{\hat{P}}_{o})\right\}}^{2}$ as an estimator of ${\mathrm{Var}}_{P_{o}}[{\dot{\tau }}_{a}(O;P_{o})-{\dot{\tau }}_{a}^{*}(O;P_{o})]$. The final analysis strategy considers the following ‘data‐adaptive’ estimator of the mean counterfactual:

$${\hat{\tau }}_{a}^{†}\;:=\left\{\begin{array}{ll} {\hat{\tau }}_{a} & \text{if}\;\hat{V}<\max\left\{n{\left({\hat{\tau }}_{a}-{\hat{\tau }}_{a}^{*}\right)}^{2}-\hat{Q},\;0\right\}+{\hat{V}}^{*}\\ {\hat{\tau }}_{a}^{*} & \text{otherwise} \end{array}\right.$$

Intuitively, in the present context, one can interpret ‘large’ values of ${\left({\hat{\tau }}_{a}-{\hat{\tau }}_{a}^{*}\right)}^{2}$ as indicating evidence against MAR Assumption 1 holding, so that the procedure selects ${\hat{\tau }}_{a}$ as the estimator. Otherwise, the procedure selects ${\hat{\tau }}_{a}^{*}$.

## 4 The Proposed Methodology in Practice

We make several observations. First, operationally, the additional data collection for those with S=1 could be achieved in a number of ways, depending on the context. In some settings, for example, it may be that telephone‐based interviews or surveys are required [21, 22]. In other instances, depending on the nature of the missing data, manual chart review or the interpretation of an image or natural language processing may be appropriate and/or sufficient [33]. Auxiliary linked adminstrative data can represent another source of the requisite additional data, for example, Cornish et al. [16] used linked educational attainment data to supplement missing outcomes in a longitudinal study of infant breastfeeding and long‐term intelligence outcomes.

Second, a crucial distinction from most prior work arises from the specific framing we have adopted; that is, that the discussions that lead to consideration of double sampling occur at the design stage of the broader study. With that framing, investigators will have substantially more control over whose data are obtained at the second stage (i.e., S) than they would over who has complete data in the EHR (i.e., R). Thus, it is important to distinguish the plausibility of MAR in the EHR from that at the second stage: while the former may be implausible due to the complexity of interactions with the health system and what measurements get recorded and when, the plausibility of the latter can be viewed as comparable with that of any prospective cohort study.

Third, the proactive nature of the proposed double‐sampling strategy provides investigators with flexibility to not only collect otherwise missing information (e.g., weight at 3 years post‐transplant) but also an opportunity to collect information that could directly inform the plausibility (or lack thereof) of the MAR assumption. If, during initial discussions (e.g., at the design stage or at the stage of writing a grant), hypotheses are put forward regarding mechanisms by which MNAR might arise, it may be possible to collect information accordingly. In the study reported by Koffman et al. [22], for example, the investigators used the survey as an opportunity to directly ask patients who had disenrolled what reasons they had for disenrolling.

Fourth, from a practical perspective, it may not be that all those who are selected actually have complete data. Settings where the data collection requires participation of the individual at‐hand will be particularly prone to this. For example, if, as in Koffman et al. [22], the mode is a telephone‐based survey, then there is no guarantee that all those who are selected will respond/engage. Other modes of ascertainment, however, such as manual chart review or the interpretation of an image, do not require direct engagement, so that it is reasonable to foresee that all those with S=1 will indeed have complete data. It's worth noting that this practical issue affects any methodology geared towards ‘additional data collection’, including outcome‐dependent sampling schemes such as the case‐control study design.

Nevertheless, for those settings where participation is required and one finds that not all individuals who are selected actually have complete data, then S can be re‐framed as an indicator of ‘selection and participation’. With this, one can still proceed with the analysis proposed in Section 3 although consideration of assumptions 2 and 3 has to be contextualized with this development. Moreover, if they can be viewed as plausibly holding, which may be the case in some settings, then one can proceed with any of the proposed analysis methods. If, however, they cannot, then alternative methods (e.g., sensitivity analyses) will be needed or the entire endeavor may need to be reconsidered.

## 5 Simulations

Table 1 provides a summary of analysis strategies #1–5 described in Sections 2.2 and 3, delineating them by the nature of the data that is taken to be available, and the assumptions that must hold for the corresponding estimator to be consistent. In this section, we present two simulation studies, conducted to investigate properties of the five strategies. In the first, we demonstrate the validity of the double sampling approach for handling MNAR data, verify the robustness properties of the proposed nonparametric influence function‐based estimator ${\hat{\tau }}_{a}$, and compare, under differing degrees of violation of MAR, the bias and variance of ${\hat{\tau }}_{a}$ to ${\hat{\tau }}_{a}^{*}$ (strategies #2 and #3, respectively) as well as approaches that only use the initially observed incomplete data (strategy #1). In the second simulation study, we compare in the absence of model misspecification the performance of strategies #2–5, over a range of possible violations of MAR. We also assess the coverage and length of the proposed confidence intervals for these estimators.

**TABLE 1:** Summary of analysis strategies #1–5.

Strategy	Data	Assumptions	Estimator
#1	(L,A,R,RY)	(1)	Standard/ad‐hoc
#2	(L,A,R,S,(R+S)Y)	(2) & (3)	IF‐based, ${\hat{\tau }}_{a}$
#3	(L,A,R,S,(R+S)Y)	(1) & (2)	IF‐based, ${\hat{\tau }}_{a}^{*}$
#4	(L,A,R,S,(R+S)Y)	(1) & (2) or (2) & (3)	${\hat{\tau }}_{a}$ or ${\hat{\tau }}_{a}^{*}$
#5	(L,A,R,S,(R+S)Y)	(1) & (2) or (2) & (3)	${\hat{\tau }}_{a}^{†}$

### 5.1 Robustness, Bias, and Variance

The framing of the simulation study is, following our motivating study in Section 2, a hypothetical study comparing two bariatric surgery procedures on long‐term weight outcomes. Specifically, we consider a binary point exposure A, taking on a value of 0 for Roux‐en‐Y gastric bypass (RYGB) and 1 for vertical sleeve gastrectomy (VSG), and continuous outcome Y of the proportion weight change at three years post‐surgery. For simplicity, we consider only one confounder, that being gender, denoted $L_{g}$. The estimand of interest is taken to be the ATE, $\tau_{1}(P_{o})-\tau_{0}(P_{o})$.

To help ground the simulation in a real‐world setting, we used information on 5693 patients who underwent either VSG or RYGB at Kaiser Permanente Washington between January 1, 2008, and December 31, 2010. For these patients, complete information was available on gender, bariatric surgery procedure, and weight outcomes, so that missingness in the outcome could then be induced by a known mechanism. We then generated 5000 simulated datasets of size n=5693 under each of three settings in which we varied the strength of the violation of MAR. Specifically, we proceeded by (i) sampling directly from the empirical distribution of $L_{g}$; (ii) generating $A|L_{g}∼\text{Bernoulli}(p_{1}L_{g}+p_{0}(1-L_{g}))$, where $p_{0}=0.20$ and $p_{1}=0.34$ were taken to approximately mirror their empirical values; (iii) generating $R|L_{g},A∼\text{Bernoulli}(\text{expit}(\delta_{0}+\delta_{L}L_{g}+\delta_{A}A+\delta_{LA}L_{g}A))$, where $\left(\delta_{0},\delta_{L},\delta_{A},\delta_{LA})=(-1.39,0.09,-0.05,-0.35\right)$ and expit(x)=exp(x)/(1+exp(x)), inducing a marginal missingness probability $P_{o}[R=0]\approx 0.8$; (iv) generating $Y|L_{g},A,R∼𝒩(\beta_{0}+\beta_{L}L_{g}+\beta_{A}A+\beta_{RA}RA,\sigma_{Y}^{2})$, where $\left(\beta_{0},\beta_{L},\beta_{A})=(-0.24,0.023,0.064\right)$, $\beta_{RA}\in \{0,0.016,0.032\}$ and $\sigma_{Y}=0.11$, approximately mirroring the marginal empirical distribution of Y; and (v) generating $S|L_{g},A,R∼\text{Bernoulli}((1-R)\text{expit}\left\{\zeta_{0}+\zeta_{L}L_{g}+\zeta_{A}A+\zeta_{LA}L_{g}A\right\})$, where $\left(\zeta_{0},\zeta_{L},\zeta_{A},\zeta_{LA})=(-2.2,0.4,0.3,0.25\right)$, inducing a marginal double sampling probability $P_{o}[S=1|R=0]\approx 0.11$. The three simulation settings differ only in the value of $\beta_{RA}$. Specifically, the parameter $\beta_{RA}$ controls the degree to which the MAR assumption is violated: when $\beta_{RA}=0.032$, we say there is a “large” violation; when $\beta_{RA}=0.016$, there is a more “moderate” violation; and, when $\beta_{RA}=0$, then there is no violation of MAR (i.e., Assumption 1 holds). The labels of “moderate” and “large” are admittedly somewhat subjective, but we use them as they qualitatively describe the distance between $\tau_{1}(P_{o})-\tau_{0}(P_{o})$, which does not assume MAR, and $\tau_{1}^{*}(P_{o})-\tau_{0}^{*}(P_{o})$, which does.

In all scenarios for $\beta_{RA}$, we computed the nonparametric influence function‐based estimator ${\hat{\tau }}_{1}-{\hat{\tau }}_{0}$, where we plugged in the maximum likelihood estimators of the true generating models $\pi_{a},\gamma_{a},\mu_{a,R},\mu_{a,S}$ described above, and assumed the double sampling probabilities $\eta_{a,0}$ were known. To verify the theoretical robustness of our influence function‐based estimator, we considered misspecifying (i) models $\left({\hat{\mu }}_{a,S},{\hat{\mu }}_{a,R},{\hat{\gamma }}_{a}\right)$, (ii) the model ${\hat{\pi }}_{a}$, and (iii) both $\left({\hat{\mu }}_{a,S},{\hat{\mu }}_{a,R},{\hat{\gamma }}_{a}\right)$ and ${\hat{\pi }}_{a}$. In particular, ${\hat{\mu }}_{a,S},{\hat{\mu }}_{a,R}$ were misspecified by omitting the main effect of $L_{g}$ (i.e., corresponding to the coefficient $\zeta_{L}$ in the data generating mechanism described above), ${\hat{\gamma }}_{a}$ by omitting the main effect of A (i.e., $\delta_{A}$) and its interaction with $L_{g}$ (i.e., $\delta_{LA}$), and ${\hat{\pi }}_{a}$, quite drastically, by estimating $P_{o}[A=a|L_{g}]$ using ${\hat{P}}_{o}[A=1-a|L_{g}]$.

For comparison, we also computed: (1) the estimator ${\hat{\tau }}_{1}^{*}-{\hat{\tau }}_{0}^{*}$, based on the influence functions ${\dot{\tau }}_{a}^{*}$ that are efficient under MAR (analysis strategy #3); and (2) estimators that did not make use of the second‐stage outcomes (analysis strategy #1). We acknowledge that there are very many approaches one might consider for analyzing the data using only the initially observed data, but decided that a reasonable analyst might assume MAR, and proceed by targeting $\xi_{1}(P_{o})-\xi_{0}(P_{o})$, where $\xi_{a}(P_{o})={𝔼}_{P_{o}}[\mu_{a,R}(L)]$, with an outcome regression based estimator based on the g‐formula (i.e., averaging $\mu_{a,R}$ over the empirical distribution of $L_{g}$), an inverse‐probability weighted (IPW) estimator with missingness‐treatment weights $\pi_{a}\gamma_{a}$, as described in Ross et al. [34], or an augmented‐IPW estimator combining both approaches as in Davidian, Tsiatis, and Leon [35] and Williamson, Forbes, and Wolfe [36]. We pitted each of these estimators (using the correct models for $\mu_{a,\text{MAR}}$, $\mu_{a,R}$, $\pi_{a}$, and $\gamma_{a}$) against our influence function‐based estimator in all three scenarios.

**FIGURE 2:**
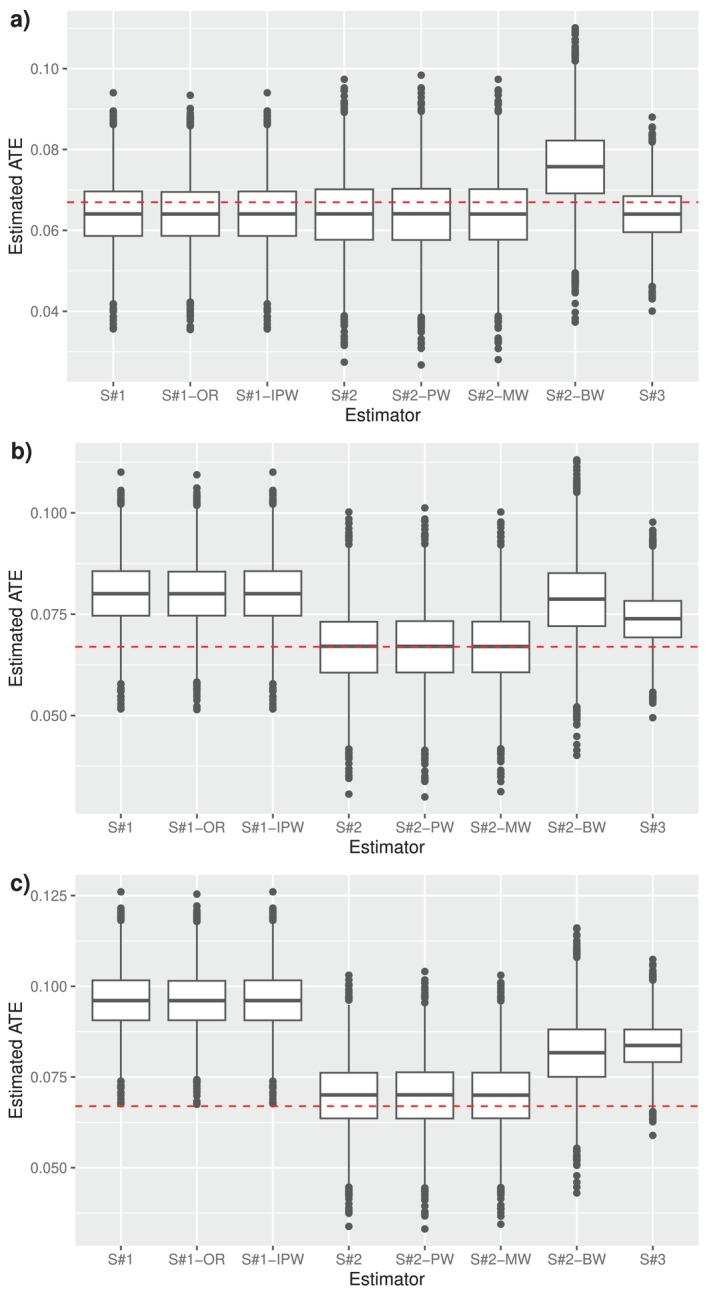
Simulation results for experiments of Section 5.1, with (a) no violation of MAR ($\beta_{RA}=0$); (b) a moderate violation of MAR ($\beta_{RA}=0.016$); and (c) a large violation of MAR ($\beta_{RA}=0.032$). S#1 = Strategy #1, augmented IPW‐based estimator assuming MAR, ${\hat{\xi }}_{1}-{\hat{\xi }}_{0}$; S#1‐OR = outcome regression‐based estimator assuming MAR; S#1‐IPW = IPW‐based estimator assuming MAR; S#2 = Strategy #2, influence‐function based estimator using double sampling, ${\hat{\tau }}_{1}-{\hat{\tau }}_{0}$; S#2‐PW = S#2 but with ${\hat{\pi }}_{a}$ misspecified; S#2‐MW = S#2‐DS but with $\left({\hat{\mu }}_{a,S},{\hat{\mu }}_{a,R},{\hat{\gamma }}_{a}\right)$ misspecified; S#2‐BW = S#2 but with both $\left({\hat{\mu }}_{a,S},{\hat{\mu }}_{a,R},{\hat{\gamma }}_{a}\right)$ and ${\hat{\pi }}_{a}$ misspecified; S#3 = Strategy #3, semiparametric efficient estimator under MAR, ${\hat{\tau }}_{1}^{*}-{\hat{\tau }}_{0}^{*}$. ATE = average treatment effect; the red dashed line indicates the true ATE.

The results of the simulation study are presented in Figure 2. The robustness of the influence‐function based estimator ${\hat{\tau }}_{1}-{\hat{\tau }}_{0}$ is clearly seen, as unbiased inference was obtained in all scenarios when all models were correctly specified, or either $\left({\hat{\mu }}_{a,S},{\hat{\mu }}_{a,R},{\hat{\gamma }}_{a}\right)$ or ${\hat{\pi }}_{a}$ was misspecified. When both were misspecified, some bias was observed in all three MAR violation scenarios. The initial‐sample‐only MAR‐based estimators had slightly lower variance, but were substantially biased when there was even a moderate violation of MAR. The MAR‐efficient estimator ${\hat{\tau }}_{1}^{*}-{\hat{\tau }}_{0}^{*}$, as expected, had the lowest variance of all estimators and was unbiased in the MAR scenario. When there was a moderate or large violation of MAR, ${\hat{\tau }}_{1}^{*}-{\hat{\tau }}_{0}^{*}$ was biased, though less so than the initial‐sample‐only MAR‐based estimators.

### 5.2 Inference and Assessment of Adaptive Estimator

Within the same simulation framework, we also assessed the performance of the adaptive estimator (analysis strategy #5), and evaluated proposed confidence intervals of all the estimators considered. For each value in a grid of MAR violation parameters $\beta_{RA}\in [0,0.04]$, we simulated 5000 datasets exactly as in the previous section. In each case, we computed both ${\hat{\tau }}_{1}-{\hat{\tau }}_{0}$ and ${\hat{\tau }}_{1}^{*}-{\hat{\tau }}_{0}^{*}$, where all underlying nuisance models were correctly specified. Based on these, we then also computed the adaptive estimator ${\hat{\tau }}_{1}^{†}-{\hat{\tau }}_{0}^{†}$.

Lastly, to show that care is required when using the data to decide between ${\hat{\tau }}_{1}-{\hat{\tau }}_{0}$ and ${\hat{\tau }}_{1}^{*}-{\hat{\tau }}_{0}^{*}$, we contrasted ${\hat{\tau }}_{1}^{†}-{\hat{\tau }}_{0}^{†}$ to an ad hoc adaptive estimator (analysis strategy #4). For this, we first test the hypothesis that $\tau_{a}(P_{o})=\tau_{a}^{*}(P_{o})$ by assessing the magnitude of the difference ${\hat{\tau }}_{a}-{\hat{\tau }}_{a}^{*}$. Formally, under appropriate conditions, Theorem A.1 implies that $\sqrt{n}({\hat{\tau }}_{a}-{\hat{\tau }}_{a}^{*})\overset{d}{\underset{\text{MAR}}{\to }}𝒩(0,Q)$, where $Q={\mathrm{Var}}_{P_{o}}({\dot{\tau }}_{a}(O;P_{o}))+{\mathrm{Var}}_{P_{o}}({\dot{\tau }}_{a}^{*}(O;P_{o}))-2\cdot {\text{Cov}}_{P_{o}}({\dot{\tau }}_{a}(O;P_{o}),{\dot{\tau }}_{a}^{*}(O;P_{o}))$. An ad hoc adaptive estimator is simply to choose ${\hat{\tau }}_{a}$ if we reject a test of MAR based on this result, that is, if $\sqrt{n}\left|{\hat{\tau }}_{a}-{\hat{\tau }}_{a}^{*}\right|/\sqrt{\hat{Q}}>z_{1-\alpha /2}$, and otherwise choose ${\hat{\tau }}_{a}^{*}$ if we fail to reject. We computed this estimator across all simulation settings.

To evaluate confidence intervals, we again focused on the three parameter values $\beta_{RA}\in \{0,0.016,0.032\}$. In the 5000 simulated datasets for each value, we constructed confidence intervals for the four estimators described above. For ${\hat{\tau }}_{1}-{\hat{\tau }}_{0}$ and ${\hat{\tau }}_{1}^{*}-{\hat{\tau }}_{0}^{*}$, we used influence function‐based Wald‐type confidence intervals. For ${\hat{\tau }}_{1}^{†}-{\hat{\tau }}_{0}^{†}$, we constructed confidence intervals based on Theorem 4 of Rothenhäusler [32]. For the ad hoc adaptive estimator, we used the Wald‐type interval corresponding to the baseline estimator chosen according to the hypothesis test—a naive approach which we expect will lead to undercoverage.

**FIGURE 3:**
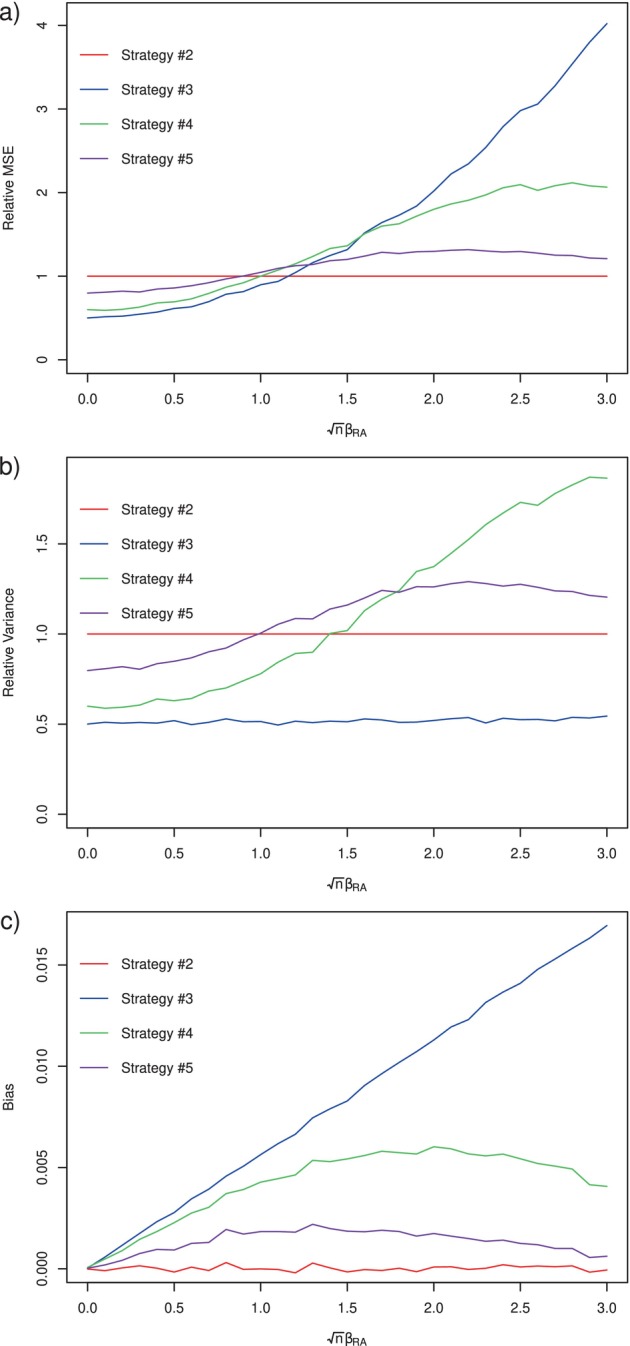
Simulation results for experiments of Section 5.2, comparing Strategy #2 of the nonparametric influence function‐based estimator (${\hat{\tau }}_{1}-{\hat{\tau }}_{0}$), Strategy #3 of the semiparametric efficient estimator under MAR (${\hat{\tau }}_{1}^{*}-{\hat{\tau }}_{0}^{*}$), Strategy #4 of the ad hoc approach described in Section 5.1, and Strategy #5 of the adaptive estimator of Rothenhäusler [32] (${\hat{\tau }}_{1}^{†}-{\hat{\tau }}_{0}^{†}$). Subplot (a) shows the empirical mean squared error (MSE) of each approach, divided by the MSE of the nonparametric efficient estimator. Subplot (b) shows the empirical variance of each approach, divided by the variance of the nonparametric efficient estimator. Subplot (c) shows the empirical bias of each approach.

The results on the grid of $\beta_{RA}$ values are shown in Figure 3. When $\beta_{RA}=0$ (i.e., MAR holds), all estimators are unbiased, ${\hat{\tau }}_{1}^{*}-{\hat{\tau }}_{0}^{*}$ is most efficient, and the two adaptive estimators have variance somewhere between that of ${\hat{\tau }}_{1}^{*}-{\hat{\tau }}_{0}^{*}$ and ${\hat{\tau }}_{1}-{\hat{\tau }}_{0}$. As $\beta_{RA}$ increases, the bias of ${\hat{\tau }}_{1}^{*}-{\hat{\tau }}_{0}^{*}$, which wrongly assumes MAR, increases roughly linearly. The two adaptive estimators also inherit some bias due to being pulled away by ${\hat{\tau }}_{1}^{*}-{\hat{\tau }}_{0}^{*}$. Interestingly, when $\beta_{RA}$ becomes really large, indicating quite a substantial violation of MAR, the bias of the two adaptive estimators returns back towards zero, as it becomes increasingly rare for either of these to select the estimator which assumes MAR.

The results on the focused set of values $\beta_{RA}\in \{0,0.016,0.032\}$ are arranged in Table 2. The confidence interval for ${\hat{\tau }}_{1}-{\hat{\tau }}_{0}$ has the appropriate coverage in all scenarios, as does the interval for ${\hat{\tau }}_{1}^{*}-{\hat{\tau }}_{0}^{*}$ when MAR holds. In the two MNAR settings, however, ${\hat{\tau }}_{1}^{*}-{\hat{\tau }}_{0}^{*}$ is biased and its confidence interval is off target. The confidence interval for the adaptive estimator ${\hat{\tau }}_{1}^{†}-{\hat{\tau }}_{0}^{†}$ also appears to be valid, with perhaps a bit of undercoverage in finite samples for moderately large values of $\beta_{RA}$. Finally, the naive confidence intervals of the ad hoc adaptive estimator tend to be overly narrow.

**TABLE 2:** Coverage and other statistics of influence function‐based and adaptive estimators.

		% Bias	Rel. variance	Rel. MSE	% Coverage	Average length
$\beta_{RA}=0$	${\hat{\tau }}_{1}-{\hat{\tau }}_{0}$	‐0.02	1.00	1.00	94.5	0.036
	${\hat{\tau }}_{1}^{*}-{\hat{\tau }}_{0}^{*}$	0.06	0.50	0.50	95.4	0.026
	${\hat{\tau }}_{1}^{†}-{\hat{\tau }}_{0}^{†}$	0.04	0.80	0.80	95.8	0.035
	Ad hoc adaptive	0.08	0.64	0.64	93.8	0.026
$\beta_{RA}=0.016$	${\hat{\tau }}_{1}-{\hat{\tau }}_{0}$	‐0.01	1.00	1.00	94.5	0.036
	${\hat{\tau }}_{1}^{*}-{\hat{\tau }}_{0}^{*}$	10.26	0.50	1.06	81.8	0.026
	${\hat{\tau }}_{1}^{†}-{\hat{\tau }}_{0}^{†}$	2.87	1.08	1.12	94.1	0.036
	Ad hoc adaptive	5.67	1.01	1.18	82.6	0.028
$\beta_{RA}=0.032$	${\hat{\tau }}_{1}-{\hat{\tau }}_{0}$	‐0.03	1.00	1.00	94.5	0.036
	${\hat{\tau }}_{1}^{*}-{\hat{\tau }}_{0}^{*}$	19.57	0.51	2.72	45.3	0.026
	${\hat{\tau }}_{1}^{†}-{\hat{\tau }}_{0}^{†}$	1.69	1.26	1.28	91.3	0.037
	Ad hoc adaptive	4.56	1.56	1.68	73.6	0.031

*Notes*: MSE, mean squared error; % Bias, $100\times \frac{\text{mean}-(\tau_{1}(P_{o})-\tau_{0}(P_{o}))}{\tau_{1}(P_{o})-\tau_{0}(P_{o})}$; Rel. variance, empirical variance of estimator divided by that of ${\hat{\tau }}_{1}-{\hat{\tau }}_{0}$; Rel. MSE, empirical MSE of estimator divided by that of ${\hat{\tau }}_{1}-{\hat{\tau }}_{0}$; % Coverage, percentage of confidence intervals across 5000 simulation replications that cover the true ATE; Average length, average confidence interval length across 5000 simulation replications.

## 6 Data Application

In this section, we present an analysis of the proposed methods to data from an EHR‐based study comparing the effect of RYGB (A=1) versus VSG (A=0) bariatric surgery procedures on percent weight change at three years post‐surgery (Y). Data were obtained from three health care sites within Kaiser Permanente: Northern California, Southern California, and Washington. Namely, in line with Arterburn et al. [23], we use data on n=13,514 adult patients who underwent RYGB or VSG between January 2005 and September 2015, with complete weight data at baseline (closest measurement pre‐surgery, up to 6 months) and follow‐up (closest measurement within ± 90 days). See Table S1 in the Supporting Information for a summary of baseline characteristics. These data comprised the “complete‐cases” from a larger collection of 30,991 patients for which follow‐up outcomes were only partially observed.

We artificially imposed missingness in the outcome Y on the complete‐case data according to an MAR mechanism, as well as an MNAR mechanism. To construct a realistic MAR mechanism, we modeled the probability of missingness from the original larger collection of 30,991 patients using the following baseline covariates L: baseline weight, health care site, year of surgery, age, gender, race/ethnicity, number of days of health care use in 7‐12 month period pre‐surgery, number of days hospitalized in pre‐surgery year, smoking status, Charlson/Elixhauser comorbidity score, insurance type, clinical statuses for hypertension, coronary artery disease, diabetes, dyslipedemia, retinopathy, neuropathy, and mental health disorders, and use of medicines including, insulin, ACE inhibitors, ARB, statins, other lipid lowering medications, and other antihypertensives. We regressed the indicator for observing Y on A and L via a SuperLearner ensemble (with library {SL.glm,SL.ranger,SL.rpart}) using the corresponding R package [37], thus obtaining $\hat{\gamma }$ satisfying MAR, that is, the probabilities of initially observing the outcome are $\hat{\gamma }\equiv P_{o}[R=1|L,A]$. Next, to construct a MNAR mechanism, we augmented the fitted values $\hat{\gamma }$ to include dependence on the outcome Y:

$$\begin{array}{ll} & \hat{\gamma }\;\mapsto \;\text{expit}\\ & \;\{(\text{logit}(\hat{\gamma })+\tilde{Y}[0.7+0.8(1-A)-1.2\;1(\text{diabetes})\;\;\;\\ & \;\;\;+0.6\;1(\text{non‐commercial insurance})])\}, \end{array}$$

where $\tilde{Y}$ is standardized BMI change at 3 years. Finally, these models were used to impose missing outcomes on the sample of n=13,514 patients by sampling R according to a Bernoulli with probability given by the fitted values from the models. The resulting marginal probabilities of missingness were 26% and 28% in the MAR and MNAR settings, respectively.

In each of the missingness settings described above, we considered collecting a random subsample (i.e., those with S=1) of initially missing outcomes of size 500, 1000, and 1500. For each of the six resulting datasets, we computed and compared point estimates and 95% confidence intervals for analysis strategies #1, #2, #3, and #5. For analysis strategy #1, we targeted $\xi_{1}(P_{o})-\xi_{0}(P_{o})$, where $\xi_{a}(P_{o})={𝔼}_{P_{o}}(\mu_{a,R}(L))$, with an augmented‐IPW estimator as in Davidian, Tsiatis, and Leon [35] and Williamson, Forbes, and Wolfe [36]. Analysis strategies #2, #3, and #5, correspond to estimators ${\hat{\tau }}_{1}-{\hat{\tau }}_{0}$, ${\hat{\tau }}_{1}^{*}-{\hat{\tau }}_{0}^{*}$, and ${\hat{\tau }}_{1}^{†}-{\hat{\tau }}_{0}^{†}$, respectively. As a benchmark, we compare to a standard full‐data augmented‐IPW estimator that uses outcome data from all n=13,514 patients. For all estimators, we used flexible SuperLearner ensembles for each component nuisance function, with a library of SL.glm, SL.ranger, and SL.rpart.

**FIGURE 4:**
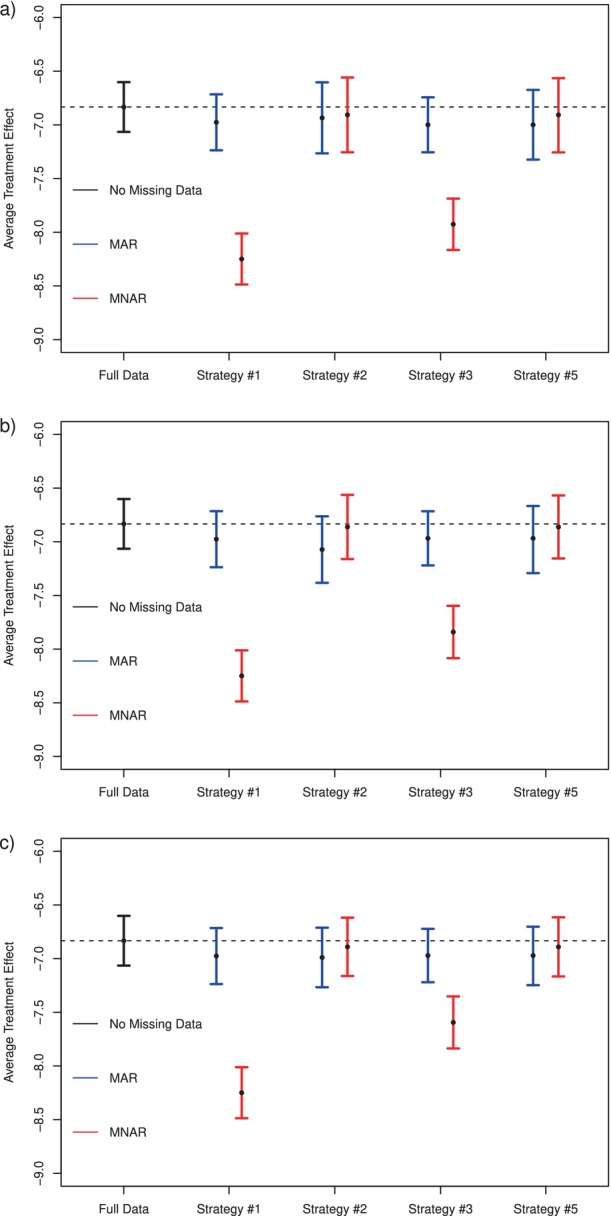
Results for data application in Section 6, showing the estimated effect on percent total weight change at 3 years. Subplots (a), (b), and (c) correspond to follow‐up subsamples of size 500, 1000, and 1500 individuals. Blue and red lines refer to estimated 95% confidence intervals when outcomes are initially MAR, and MNAR, respectively. Point estimates are marked with black dots. Strategy #1 = augmented‐IPW estimator with incomplete data only, assuming MAR; Strategy #2 = influence‐function based estimator using double sampling, ${\hat{\tau }}_{1}-{\hat{\tau }}_{0}$; Strategy #3 = semiparametric efficient estimator under MAR, ${\hat{\tau }}_{1}^{*}-{\hat{\tau }}_{0}^{*}$; Strategy #5 = adaptive estimator of Rothenhäusler [32], ${\hat{\tau }}_{1}^{†}-{\hat{\tau }}_{0}^{†}$. The black dashed line represents the point estimate for the benchmark full data analysis.

Results are summarized in Figure 4. When MAR holds, all estimators perform well with respect to the benchmark analysis, with ${\hat{\tau }}_{1}^{*}-{\hat{\tau }}_{0}^{*}$ having smallest variance, as anticipated. On the other hand, the estimators that assume MAR appear to be biased under MNAR, while the nonparametric efficient estimator ${\hat{\tau }}_{1}-{\hat{\tau }}_{0}$ and adaptive estimator ${\hat{\tau }}_{1}^{†}-{\hat{\tau }}_{0}^{†}$ (which do not assume MAR) are robust to this violation of MAR. As expected, as the second stage subsample size increases, precision improves for analysis strategies #2, #3, and #5, which incorporate this data.

## 7 Discussion

In brief, this article proposes a set of substantive assumptions and statistical methods for the use of double‐sampling as a means to address potentially MNAR missing data, when scientific interest lies in estimating causal ATEs. Key to our contributions are a suite of novel analysis strategies that exploit data arising from the double sampling scheme, coupled with identifying assumptions that guarantee the corresponding estimators to be asymptotically normal, efficient, and robust.

To summarize and highlight our main empirical and theoretical findings, consider now being faced with partially missing outcomes as in the hypothetical EHR‐based study of Section 2. If one is determined not to allocate budget for additional (i.e., second stage) data collection, then one may proceed with an MAR‐based analysis using the initially observed incomplete data (Strategy #1), though there is potential for substantial bias when the MAR assumption is violated (see e.g., Figure 2b,c). Alternatively, one may be content with computing nonparametric bounds or conducting a sensitivity analysis to be more robust to potential violations of MAR. These approaches have downsides: (i) under no assumptions about the missing data structure, nonparametric bounds are necessarily very wide, and (ii) while sensitivity analyses can provide confidence sets that are robust to certain degrees of violation of MAR [9], there may be no data to support the actual value of the magnitude of this violation, making it difficult to draw actionable conclusions. If instead one is able to obtain second‐stage follow‐up data, several more options become available: nonparametric estimation based on Assumption 2 (Strategy #2), semiparametric estimation assuming the initially observed outcomes are MAR (Strategy #3), or an adaptive approach that selects between the latter two based on evidence of MAR at the first stage (Strategies #4 and #5). If one is certain that the initially observed outcomes are MAR, then Strategy #3 is valid and semiparametric efficient (see Theorem 4). In fact, in our simulations, the estimator under Strategy #3 had minimal mean squared error even under some small violations of MAR (see e.g., Figure 3a). Otherwise, if one is unsure whether MAR held, we recommend that one opt for Strategy #2 or #5 (we would rule out Strategy #4 due to the lack of associated inference tools). The nonparametric efficient estimator (Strategy #2) represents a good default choice, as it has favorable theoretical properties (see Theorem 2), and in all simulation scenarios is unbiased and its confidence intervals attained nominal coverage (see e.g., Table 2). For small or absent MAR violations, the adaptive estimator (Strategy #5) can be more efficient, but is less efficient than Strategy #1 for moderate violations (see e.g., Figure 3a).

The methods we have discussed focus on the specific causal problem outlined in Section 2. That said, the nonparametric identification and estimation results are entirely generic, and do not depend on either the data structure of the given problem nor the specific mean counterfactual estimand of interest. In Appendix A of the Supporting Information, we lay out the notation for arbitrary coarsening [38] of a given full data structure and show that under a generalization of Assumptions 2 and 3, double sampling identifies the complete data distribution; derive a transformation of the full data nonparametric influence function of an arbitrary smooth functional that yields the observed data nonparametric influence function; construct influence function‐based estimators using sample splitting; and characterize the asymptotic behavior of these estimators, including multiple robustness properties. The general coarsening formulation is powerful, and justifies methodology—analogous to the approaches described in the main paper—that are applicable for many estimands in settings with more complicated missing data patterns, right‐censoring and/or truncation (e.g., as occurs often in survival analysis settings), and beyond.

Table 1 emphasizes that each of the proposed estimators require Assumption 2 to hold. In any given applied setting, this assumption will need to be carefully evaluated. As indicated in Section 3.2, depending on the context, one practical issue is that selection by the double sampling scheme may not necessarily yield complete data. In such settings, investigators will need to work through the same thought experiments that one usually would for the standard MAR assumption (such as Assumption 2) to try to understand why some individuals engage and others do not. If it is felt that engagement remains dependent on outcome status (beyond what is explained by what is known about the design and covariates (A, L)), then sensitivity analyses or alternative identification schemes may be necessary; this is an on‐going area of our work.

A second practical issue is that, even if all those selected actually engage, the data that arises from the double‐sampling scheme may be subject to error or recall bias [21]. In some settings, the potential for recall bias may be mitigated through the design. In Koffman et al. [22], for example, the outcome of interest was weight change at five years post‐surgery, so the investigators timed the invitation to participants to coincide with the five‐year anniversary. Additionally, following the same broad philosophy of this paper, one could directly learn about potential recall bias by including some participants for whom R = 1 in the double‐sampling scheme. This, in turn, would enable a comparison between information provided by the patient and what is available in the EHR. How best to do this and use the resulting information, though, are open questions.

We also mention that, while we believe the simulations and case study illustrated in finite samples the theoretical properties we have developed for the various approaches, they were necessarily fairly limited in scope. A larger simulation study, comparing a wider variety of scenarios and component modeling strategies, will be a goal for future research.

Notwithstanding these practical issues and the fact that logistical or financial considerations may altogether preclude the use of double sampling in some settings, we believe the proposed methodology presents new options for researchers as they contend with potentially informative missing or coarsened data. Beyond those mentioned above, there are many opportunities for future work in this vein, including how best to use the available information in the EHR when allocating resources for double‐sampling, as well as developing estimators for a broader set of analysis goals, such as mediation, and outcome types, such as time‐to‐event outcomes.

## Conflicts of Interest

The authors declare no conflicts of interest.

## Supporting information




**Data S1.** Supporting Information.

**Data S2.** Supporting Information.

## Data Availability

Research data are not shared.
