# Supplementary material for: Double Sampling for Informatively Missing Data in Electronic Health Record‐Based Comparative Effectiveness Research
Source: Stat Med. 2024 Dec 5;43(30):6086–98. doi: 10.1002/sim.10298 (PMC11639654; doi:10.1002/sim.10298)
Supplement: Supplementary file 1 — Data S1. Supporting Information. [file SIM-43-6086-s001.pdf]

Supplementary Material for "Double sampling for informatively missing data in electronic health record-based comparative effectiveness research"

Alexander W. Levis, Rajarshi Mukherjee, Rui Wang,
Heidi Fischer, Sebastien J.P.A. Haneuse

July 11, 2024

A General Coarsening Framework and Nonparametric Results

A.1 Coarsened data and nonparametric identification

Suppose the desired *complete* data for a given problem are the random vector $\boldsymbol{X} \sim P^*_{\boldsymbol{X}}$. That is, with $\boldsymbol{X}$ observed on every subject in a random sample, a parameter of interest, say $\chi(P^*_{\boldsymbol{X}})$, could be estimated consistently. Suppose, however, that the *initially observed* data consists only of $(C, \sigma_C(\boldsymbol{X}))$, where $C \in \mathbb{N}$ is a coarsening random variable, and $\sigma_C(\boldsymbol{X})$ is a coarsened version of the complete data: σ_k is some (typically many-to-one) function for every possible value k of C. As in Tsiatis (2007), we will write $C = \infty$ to denote that the complete data are observed, i.e., there is no coarsening. We will further assume that there exist functions $\overline{\sigma}_k$ for every k, such that $(\sigma_k, \overline{\sigma}_k)$ is injective; that is, there exist functions h_k with $h_k(\sigma_k(\boldsymbol{X}), \overline{\sigma}_k(\boldsymbol{X})) = \boldsymbol{X}$. In our previous observational example from Section 2, $C = R \cdot \infty$, where $0 \cdot \infty = 0$, and $\sigma_0(\boldsymbol{X}) = (\boldsymbol{L}, A)$, $\overline{\sigma}_0(\boldsymbol{X}) = Y$.

We now suppose that a subsample is intensively followed up, and the initially unobserved data $\overline{\sigma}_C(\boldsymbol{X})$ are obtained on some subjects. Let $S \in \{0, 1\}$ indicate successful follow-up in the subsample when $S = 1$. The *full* data are $(C, \sigma_C(\boldsymbol{X}), S, \overline{\sigma}_C(\boldsymbol{X})) \sim P^*$, and the *final observed* data are independent and identically distributed copies of $O = (C, \sigma_C(\boldsymbol{X}), S, S \cdot \overline{\sigma}_C(\boldsymbol{X})) \sim P$. Here, as before, the observed data probability distribution P and the complete data distribution $P^*_{\boldsymbol{X}}$ are induced by P^*. Henceforth, we suppose that the data at hand are a random sample $O_1, \ldots, O_n \overset{\text{iid}}{\sim} P$.

Let $p = \frac{dP}{d\mu}$ and $p^* = \frac{dP^*}{d\mu}$ denote the densities for P and P^*, respectively, both with respect to some dominating measure μ. The density of the full data distribution can be factored via $p^*(C, \sigma_C(\boldsymbol{X}), S, \overline{\sigma}_C(\boldsymbol{X})) = p(C, \sigma_C(\boldsymbol{X}), S) \times \prod_k p^*(\overline{\sigma}_k(\boldsymbol{X}) \mid C = k, \sigma_k(\boldsymbol{X}), S)^{\mathbb{1}(C=k)}$, whereas the density of the observed data can be factored via $p(O) = p(C, \sigma_C(\boldsymbol{X}), S) \times \prod_k p(\overline{\sigma}_k(\boldsymbol{X}) \mid C = k, \sigma_k(\boldsymbol{X}), S = 1)^{\mathbb{1}(S=1, C=k)}$. As the conditioning event $S = 0$ is possible in the full data but not in the observed data (i.e., appears in expression for p^* but not for p), P^* will not be identified from the observed data distribution P unless further assumptions are made. That said, analysis of the components of the full data density p^* that are not present in p motivates the following conditions, which generalize Assumptions 2 and 3.

Assumption A.1 (No informative second-stage selection, general version)**.** *For all $k \neq \infty$, $S \perp\!\!\!\perp \overline{\sigma}_k(\boldsymbol{X}) \mid C = k, \sigma_k(\boldsymbol{X})$.*

Assumption A.2 (Positivity of second-stage sampling probabilities, general version)**.** *For some $\epsilon > 0$, $P\left[P[S = 1 \mid C = k, \sigma_k(\boldsymbol{X})] \geq \epsilon\right] = 1$, for all $k \neq \infty$.*

By the following result, a generalization of Proposition 1, Assumptions A.1 and A.2 are sufficient to identify the full data distribution P^*.

Proposition A.1. *Assumptions A.1 and A.2 are sufficient to identify the full data distribution P^* from the observed data distribution P.*

Proof. By Assumption A.1, the full data distribution may be factorized via

$$\begin{aligned} p^*(C, \sigma_C(\boldsymbol{X}), S, \overline{\sigma}_C(\boldsymbol{X})) &= p(C, \sigma_C(\boldsymbol{X}), S) p^*(\overline{\sigma}_C(\boldsymbol{X}) \mid C, \sigma_C(\boldsymbol{X}), S) \\ &= p(C, \sigma_C(\boldsymbol{X}), S) p(\overline{\sigma}_C(\boldsymbol{X}) \mid C, \sigma_C(\boldsymbol{X}), S = 1) \end{aligned} \tag{1}$$

which only depends on the observed data distribution p. Note that we may safely introduce $S = 1$ in the conditioning event due to Assumption A.2, and we may ignore the vacuous case $C = \infty$ as $\sigma_\infty(\boldsymbol{X}) = \boldsymbol{X}$, given which $\overline{\sigma}_\infty(\boldsymbol{X})$ is degenerate. □

□

The identifying Assumption A.1 may be interpreted as asserting that whether or not a subject is successfully double sampled is independent of all the initially unobserved data, conditional on all the initially observed data. While we have circumvented the need for the usual coarsening at random assumption for the initial sample, it is worth noting that given only observed data O, Assumption A.1 is untestable. In practice, however, these can be ensured by certain study designs and the successful follow-up of the chosen subsample: (i) subsample selection completely at random prior to the study; (ii) subsample selected at random among those with any initially missing information; and (iii) subsample selected with investigator-defined probabilities depending only on $(C, \sigma_C(\boldsymbol{X}))$. Of course, successful follow-up of the entire intended subsample may not be possible, and in these cases it must be that initially observed data is sufficient to predict successful follow-up. In general, if the same method of contacting subjects is used at the first stage and second stage of data collection, then it may be unreasonable to assume that coarsening at random fails to hold but Assumption A.1 is valid. Thus, the double sampling approach may be most justifiable when the method of data collection at the second stage differs from the first, e.g., in the EHR example, where the initial sample is the data that happened to be recorded in the electronic record, and the subsample is followed up via telephone or in-depth chart review.

On the other hand, Assumption A.2 asserts that there are no subpopulations, defined by observed data patterns, that are systematically excluded from the double sampling strategy (other than those with initially complete data) — if this were not the case, one could not learn about the subpopulations with initial missing information that were not followed up.

A.2 Estimation of complete data parameters

Suppose interest lies in estimating the complete data parameter $\chi(P^*_{\boldsymbol{X}}) \in \mathbb{R}$, viewed as a functional from a model space of probability distributions on $\boldsymbol{X}$ — to which $P^*_{\boldsymbol{X}}$ belongs — to the real line. By Proposition A.1, under Assumptions A.1 and A.2, any complete data functional $\chi(P^*_{\boldsymbol{X}})$ has a corresponding observed data functional representation $\tau(P)$. For example, if $\chi(P^*_{\boldsymbol{X}}) = \mathbb{E}_{P^*}(g(\boldsymbol{X}))$, for some function g, then $\chi(P^*_{\boldsymbol{X}}) = \mathbb{E}_P(\mathbb{E}_{P^*}(g(\boldsymbol{X}) \mid C, \sigma_C(\boldsymbol{X}))) = \mathbb{E}_P(\mathbb{E}_P(g(\boldsymbol{X}) \mid C, \sigma_C(\boldsymbol{X}), S = 1)) =: \tau(P)$. It will often be the case that if the complete data $\boldsymbol{X}$ were completely observed, one would have in mind a valid estimator of $\chi(P^*_{\boldsymbol{X}})$. A natural goal is thus to develop a general procedure that can in a certain sense transform a complete-data estimator into one that uses only the observed data $O_1, \ldots, O_n$. The following proposition is the key semiparametric-theoretical result that will facilitate such

a procedure. Note that it can be seen as a special case of the general theory developed in Robins et al. (1994).

Proposition A.2. *Suppose* χ *is pathwise differentiable* [1] *with respect to the complete data model at* $P^*_{\boldsymbol{X}}$*, with influence function* $\dot\chi(\boldsymbol{X}; P^*_{\boldsymbol{X}})$ *(with respect to maximal tangent space), and that Assumptions A.1 and A.2 hold. Then* $\tau(P) = \chi(P^*_{\boldsymbol{X}})$ *is pathwise differentiable with influence function* $\dot\tau(O;P) = \nu_C(\sigma_C(\boldsymbol{X})) + \frac{S}{\eta(C,\sigma_C(\boldsymbol{X}))}\{\dot\chi(\boldsymbol{X};P^*_{\boldsymbol{X}}) - \nu_C(\sigma_C(\boldsymbol{X}))\}$ *at* P*, where* $\nu_C(\sigma_C(\boldsymbol{X})) = \mathbb{E}_P(\dot\chi(\boldsymbol{X};P^*_{\boldsymbol{X}}) \mid C, \sigma_C(\boldsymbol{X}), S = 1)$*, and* $\eta(C,\sigma_C(\boldsymbol{X})) = P[S = 1 \mid C, \sigma_C(\boldsymbol{X})]$*. Recalling that* $\sigma_\infty(\boldsymbol{X}) = \boldsymbol{X}$*, we allow for the possibility that* $\eta(\infty, \boldsymbol{X}) = 0$*, and define* $\dot\tau(O;P)$ *to equal* $\nu_\infty(\boldsymbol{X}) = \dot\chi(\boldsymbol{X};P^*_{\boldsymbol{X}})$ *when* $C = \infty$*.*

Proof. Recall from Bickel et al. (1993) and Tsiatis (2007) that an *influence function* of a pathwise differentiable functional $\chi(P)$, at P in a given statistical model, is a zero-mean finite-variance function $\dot\chi(O;P)$ of observed data O such that for any regular parametric submodel $\{P_\epsilon : \epsilon \in [0,1)\}$ through $P_0 \equiv P$, it holds that

$$\frac{d}{d\epsilon}\chi(P_\epsilon)\bigg|_{\epsilon=0} = \mathbb{E}_P\left(\dot\chi(O;P)g(O)\right),$$

where $g(O)$ is the score function of the parametric submodel at P. The *tangent set* $\mathcal{T}_P$ of the statistical model at P is the set of all score functions of one-dimensional regular parametric submodels through P, and the *tangent space* is $\Lambda_P = \overline{[\mathcal{T}_P]}$, the closure of the linear span (with respect to the Hilbert space $L_2(P)$) of the tangent set. The *efficient influence function* of χ at P is the unique influence function belonging to Λ_P. When $\Lambda_P = L_2(P)$, the model is said to be nonparametric, and there is a unique influence function, often called the nonparametric influence function. See Bickel et al. (1993) and Van der Vaart (2000) for precise definitions.

Let $\{P_\epsilon \mid \epsilon \in [0,1)\}$ be an arbitrary one-parameter regular parametric submodel through $P \equiv P_0$. Note that, for any $\epsilon \in [0,1)$,

$$p_\epsilon(O) = p_\epsilon(C, \sigma_C(\boldsymbol{X}), S)p_\epsilon(\overline{\sigma}_C(\boldsymbol{X}) \mid C, \sigma_C(\boldsymbol{X}), S = 1)^S,$$

so we must have

$$g(O) = g_{C,\sigma_C(\boldsymbol{X}),S} + S \cdot g_{\overline{\sigma}_C(\boldsymbol{X})|C,\sigma_C(\boldsymbol{X}),S=1},$$

where $g(O)$ is the score function of the submodel, typically

$$g(O) = \frac{d}{d\epsilon}\log\left(p_\epsilon(O)\right)\bigg|_{\epsilon=0},$$

and $g_{A|B}$ is the conditional score of A given B for arbitrary variables A, B. Of course, this submodel defines a regular parametric submodel $\{P^*_\epsilon \mid \epsilon \in [0,1)\}$ through the full data distribution $P^* \equiv P^*_0$ by Assumption A.1: for any $\epsilon \in [0,1)$,

$$p^*_\epsilon(C, \sigma_C(\boldsymbol{X}), S, \overline{\sigma}_C(\boldsymbol{X})) = p_\epsilon(C, \sigma_C(\boldsymbol{X}), S)p_\epsilon(\overline{\sigma}_C(\boldsymbol{X}) \mid C, \sigma_C(\boldsymbol{X}), S = 1).$$

Next, observe that

$$\begin{aligned}
&\mathbb{E}_P\left(\frac{S}{\eta(C,\sigma_C(\boldsymbol{X}))}\nu_C(\sigma_C(\boldsymbol{X}))g(O)\right)\\
&= \mathbb{E}_P\left(\frac{S}{\eta(C,\sigma_C(\boldsymbol{X}))}\nu_C(\sigma_C(\boldsymbol{X}))g_{C,\sigma_C(\boldsymbol{X}),S}\right),\\
&= \mathbb{E}_P\left(\frac{S}{\eta(C,\sigma_C(\boldsymbol{X}))}\dot\chi(\boldsymbol{X};P^*_{\boldsymbol{X}})g_{C,\sigma_C(\boldsymbol{X}),S}\right).
\end{aligned}$$

[1] see e.g. Bickel et al. (1993, Chapter 3) for precise definitions.

Here, the first equality results from conditioning on $(C, \sigma_C(\boldsymbol{X}), S)$, given which $g_{\overline{\sigma}_C(\boldsymbol{X})|C,\sigma_C(\boldsymbol{X}),S}$ has mean zero ($g_{\overline{\sigma}_C(\boldsymbol{X})|C,\sigma_C(\boldsymbol{X}),S=1}$ can be replaced by $g_{\overline{\sigma}_C(\boldsymbol{X})|C,\sigma_C(\boldsymbol{X}),S}$ due to the presence of indicator S). The second equality again can be seen by conditioning throughout by $(C, \sigma_C(\boldsymbol{X}), S)$. Thus,

$$
\begin{aligned}
&\mathbb{E}_P\left(\frac{S}{\eta(C, \sigma_C(\boldsymbol{X}))}\left\{\dot{\chi}(\boldsymbol{X}; P^*_{\boldsymbol{X}}) - \nu_C(\sigma_C(\boldsymbol{X}))\right\} g(O)\right) \\
&= \mathbb{E}_P\left(\frac{S}{\eta(C, \sigma_C(\boldsymbol{X}))}\dot{\chi}(\boldsymbol{X}; P^*_{\boldsymbol{X}})[g(O) - g_{C,\sigma_C(\boldsymbol{X}),S}]\right), \\
&= \mathbb{E}_P\left(\frac{S}{\eta(C, \sigma_C(\boldsymbol{X}))}\dot{\chi}(\boldsymbol{X}; P^*_{\boldsymbol{X}}) g_{\overline{\sigma}_C(\boldsymbol{X})|C,\sigma_C(\boldsymbol{X}),S=1}\right), \text{ since } S^2 = S, \\
&= \mathbb{E}_{P^*}\left(\frac{P^*[S = 1 \mid C, \sigma_C(\boldsymbol{X}), \overline{\sigma}_C(\boldsymbol{X})]}{\eta(C, \sigma_C(\boldsymbol{X}))}\dot{\chi}(\boldsymbol{X}; P^*_{\boldsymbol{X}}) g_{\overline{\sigma}_C(\boldsymbol{X})|C,\sigma_C(\boldsymbol{X}),S=1}\right), \\
&= \mathbb{E}_{P^*}\left(\dot{\chi}(\boldsymbol{X}; P^*_{\boldsymbol{X}}) g_{\overline{\sigma}_C(\boldsymbol{X})|C,\sigma_C(\boldsymbol{X}),S=1}\right).
\end{aligned}
$$

In the third equality, we introduced P^* as it induces P, we conditioned on $(C, \sigma_C(\boldsymbol{X}), \overline{\sigma}_C(\boldsymbol{X}))$, and used the fact that, by construction, $\boldsymbol{X}$ is equal to $h_C(\sigma_C(\boldsymbol{X}), \overline{\sigma}_C(\boldsymbol{X}))$; in the fourth equality, we used Assumption A.1.

Now, see that

$$
\begin{aligned}
\mathbb{E}_P\left(\nu_C(\sigma_C(\boldsymbol{X})) g(O)\right) &= \mathbb{E}_P\left(\nu_C(\sigma_C(\boldsymbol{X})) g_{C,\sigma_C(\boldsymbol{X}),S}\right), \\
&= \mathbb{E}_{P^*}\left(\dot{\chi}(\boldsymbol{X}; P^*_{\boldsymbol{X}}) g_{C,\sigma_C(\boldsymbol{X}),S}\right),
\end{aligned}
$$

where in the first equality we note that $S \cdot g_{\overline{\sigma}_C(\boldsymbol{X})|C,\sigma_C(\boldsymbol{X}),S=1}$ has mean zero given $(C, \sigma_C(\boldsymbol{X}), S)$, and the second equality can be seen by conditioning on $(C, \sigma_C(\boldsymbol{X}), S)$ and again using $S \perp\!\!\!\perp \overline{\sigma}_C(\boldsymbol{X}) \mid C, \sigma_C(\boldsymbol{X})$ under P^*.

Finally, defining

$$
\dot{\tau}(O; P) = \nu_C(\sigma_C(\boldsymbol{X})) + \frac{S}{\eta(C, \sigma_C(\boldsymbol{X}))}\left\{\dot{\chi}(\boldsymbol{X}; P^*_{\boldsymbol{X}}) - \nu_C(\sigma_C(\boldsymbol{X}))\right\},
$$

we have shown that

$$
\mathbb{E}_P\left(\dot{\tau}(O; P) g(O)\right) = \mathbb{E}_{P^*}\left(\dot{\chi}(\boldsymbol{X}; P^*_{\boldsymbol{X}}) g^*\right),
$$

where $g^* = g_{C,\sigma_C(\boldsymbol{X}),S} + g_{\overline{\sigma}_C(\boldsymbol{X})|C,\sigma_C(\boldsymbol{X}),S=1}$ is the score of the full data submodel through P^*. But by assumption that χ is regular at $P^*_{\boldsymbol{X}}$,

$$
\mathbb{E}_{P^*}\left(\dot{\chi}(\boldsymbol{X}; P^*_{\boldsymbol{X}}) g^*\right) = \left.\frac{d}{d\epsilon}\chi(P^*_{\boldsymbol{X},\epsilon})\right|_{\epsilon=0},
$$

so that

$$
\left.\frac{d}{d\epsilon}\tau(P_\epsilon)\right|_{\epsilon=0} = \mathbb{E}_P\left(\dot{\tau}(O; P) g(O)\right)
$$

as $\tau(P_\epsilon) = \chi(P^*_{\boldsymbol{X},\epsilon})$ for all $\epsilon \in [0, 1)$, by construction. By definition, this means that $\dot{\tau}(O; P)$ is an influence function for τ at P, as claimed. □

We remark that another way to interpret the term $\frac{S}{\eta(C,\sigma_C(\boldsymbol{X}))}$ in the case that $\eta(\infty, \boldsymbol{X}) = 0$ is to use the convention $0 \cdot \infty = 0$, so that the second term drops out.

We are now equipped to define a one-step estimator of the general functional $\tau(P)$ that uses its estimated influence function to correct the bias of a plugin estimator $\tau(\widehat{P})$. Depending

on the form of the parameter and its influence function, certain components of the observed data distribution may not need to be estimated (e.g., the running causal example of this paper). In general, though, an estimate of $P^*_{\boldsymbol{X}}$ can be reconstructed by marginalizing an estimated version of the identified full data density (1) over (C, S). Letting $\lambda_C(\overline{\sigma}_C(\boldsymbol{X}); \sigma_C(\boldsymbol{X}))$ be the distribution function of $\overline{\sigma}_C(\boldsymbol{X})$ given $C, \sigma_C(\boldsymbol{X}), S = 1$, we can use $\widehat{P}^*_{\boldsymbol{X}}$, $\widehat{\eta}$, $\widehat{\lambda}$ to obtain $\dot{\tau}(O; \widehat{P}) = \widehat{\nu}_C(\sigma_C(\boldsymbol{X})) + \frac{S}{\widehat{\eta}(C, \sigma_C(\boldsymbol{X}))}\left\{\dot{\chi}(\boldsymbol{X}; \widehat{P}^*_{\boldsymbol{X}}) - \widehat{\nu}_C(\sigma_C(\boldsymbol{X}))\right\}$, where $\widehat{\nu}_C(\sigma_C(\boldsymbol{X})) = \int \dot{\chi}(h_C(\sigma_C(\boldsymbol{X}), \boldsymbol{t}); \widehat{P}^*_{\boldsymbol{X}})\, d\widehat{\lambda}_C(\boldsymbol{t}; \sigma_C(\boldsymbol{X}))$.

We propose to use sample splitting and cross-fitting (Chernozhukov et al., 2018), and fit $\widehat{P}_k$ using data I_k^c, for $k = 1, \ldots, K$. We then define $\widehat{\tau}_k = \tau(\widehat{P}_k) + \frac{K}{n}\sum_{i \in I_k} \dot{\tau}(O_i; \widehat{P}_k)$, for $k = 1, \ldots, K$, so that the sample-split influence function-based estimator is given by $\widehat{\tau} = \frac{1}{K}\sum_{k=1}^{k} \widehat{\tau}_k$.

A.3 Consistency, asymptotic normality, and robustness

The following result is the basis for consistency, asymptotic normality, nonparametric efficiency, and multiple robustness of the proposed nonparametric influence function-based estimator $\widehat{\tau}$.

Theorem A.1. *Suppose* $\left\|\dot{\tau}(\,\cdot\,; \widehat{P}_k) - \dot{\tau}(\,\cdot\,; P)\right\| = o_P(1)$ *for* $k = 1, \ldots, K$. *Then*

$$\widehat{\tau} - \tau(P) = O_P\left(\frac{1}{\sqrt{n}} + \frac{1}{K}\sum_{k=1}^{K} \mathrm{Bias}_\tau(\widehat{P}_k; P)\right),$$

where $\mathrm{Bias}_\tau(\widehat{P}; P) = \mathbb{E}_P(\dot{\tau}(O; \widehat{P})) + \tau(\widehat{P}) - \tau(P)$ *for any* $\widehat{P}$. *Moreover, if* $\mathrm{Bias}_\tau(\widehat{P}_k; P) = o_P(n^{-1/2})$, *for* $k = 1, \ldots, K$, *then* $\sqrt{n}(\widehat{\tau} - \tau(P)) \xrightarrow{d} \mathcal{N}(0, V)$, *where* $V = \mathrm{Var}_P(\dot{\tau}(O; P))$ *is the nonparametric efficiency bound.*

Proof. For a given subset $k \in \{1, \ldots, K\}$, we can decompose the error of $\widehat{\tau}_k$ relative to $\tau(P)$ via:

$$\begin{aligned}
\widehat{\tau}_k - \tau(P) &= \frac{K}{n}\sum_{i \in I_k} \dot{\tau}(O_i, P) \\
&\quad + \left\{\mathbb{E}_P(\dot{\tau}(O; \widehat{P}_k)) + \tau(\widehat{P}_k) - \tau(P)\right\} \\
&\quad + \left\{\frac{K}{n}\sum_{i \in I_k}\left[\dot{\tau}(O_i; \widehat{P}_k) - \dot{\tau}(O_i, P)\right] - \mathbb{E}_P(\dot{\tau}(O; \widehat{P}_k))\right\}.
\end{aligned}$$

Thus, the error of $\widehat{\tau}$ with respect to $\tau(P)$ can be decomposed as follows:

$$\begin{aligned}
\widehat{\tau} - \tau(P) &= \frac{1}{n}\sum_{i=1}^{n} \dot{\tau}(O_i, P) + \frac{1}{K}\sum_{k=1}^{K} \mathrm{Bias}_\tau(\widehat{P}_k; P) \\
&\quad + \frac{1}{K}\sum_{k=1}^{K}\left\{\frac{K}{n}\sum_{i \in I_k}\left[\dot{\tau}(O_i; \widehat{P}_k) - \dot{\tau}(O_i, P)\right] - \mathbb{E}_P(\dot{\tau}(O; \widehat{P}_k))\right\}.
\end{aligned}$$

By the central limit theorem, the first term is $O_P(n^{-1/2})$ as

$$\frac{1}{\sqrt{n}}\sum_{i=1}^{n} \dot{\tau}(O_i, P) \xrightarrow{d} \mathcal{N}(0, V),$$

where $V = \text{Var}_P(\dot{\tau}(O;P))$. Next, invoking Lemma 2 in Kennedy et al. (2020) or Kennedy (2020), for each $k = 1, \ldots, K$, as $\{O_i : i \in I_k\} \perp\!\!\!\perp \{O_i : i \in I_k^c\}$,

$$\begin{aligned}
&\frac{K}{n} \sum_{i \in I_k} \left[\dot{\tau}(O_i; \widehat{P}_k) - \dot{\tau}(O_i, P) \right] - \mathbb{E}_P(\dot{\tau}(O; \widehat{P}_k)) \\
&= O_P \left(\frac{\sqrt{K}}{\sqrt{n}} \left\| \dot{\tau}(\,\cdot\,; \widehat{P}_k) - \dot{\tau}(\,\cdot\,; P) \right\| \right), \\
&= o_P \left(\frac{\sqrt{K}}{\sqrt{n}} \right),
\end{aligned}$$

as $n/K \to \infty$, since $\left\| \dot{\tau}(\,\cdot\,; \widehat{P}_k) - \dot{\tau}(\,\cdot\,; P) \right\| = o_P(1)$ by assumption. Equivalently, as $n \to \infty$, this k-th term is $o_P(n^{-1/2})$. As K is fixed, an average of K such terms is also $o_P(n^{-1/2})$, and the result follows. □

It is important to note that we have only established that $\widehat{\tau}$ is fully (semiparametric) efficient in a nonparametric model, i.e., when the model tangent space (see Bickel et al. (1993), Tsiatis (2007)) is $L_2^0(P)$, consisting of all mean-zero functions of O with finite variance. Nonparametric efficiency of $\widehat{\tau}$ is guaranteed because there is a unique (thus efficient) influence function in a nonparametric model, its variance equal to the nonparametric efficiency bound (Bickel et al., 1993). Derivation of the semiparametric efficient observed data influence function in proper semiparametric models where restrictions are placed on P will depend on the form of those restrictions (e.g., when MAR holds as in Analysis #3), so we leave characterizations of efficiency over classes of restrictions on P for future research.

The next two propositions relate the asymptotic variance and bias term (as defined in Proposition A.2) of $\widehat{\tau}$ to that of a complete-data influence function based estimator.

Proposition A.3. *Let $\dot{\tau}(O;P)$ be the observed data influence function defined in Proposition A.2, where P is induced by P^* satisfying Assumptions A.1 and A.2. Then the observed data nonparametric efficiency bound for estimating $\tau(P) = \chi(P_{\boldsymbol{X}}^*)$ is given by:* $\text{Var}_P(\dot{\tau}(O;P)) = \text{Var}_{P^*}(\dot{\chi}(\boldsymbol{X}; P_{\boldsymbol{X}}^*)) + \mathbb{E}_P\left(\left(\frac{1}{\eta(C, \sigma_C(\boldsymbol{X}))} - 1 \right) \text{Var}_P(\dot{\chi}(\boldsymbol{X}; P_{\boldsymbol{X}}^*) \mid C, \sigma_C(\boldsymbol{X}), S = 1) \right)$.

Proof. Note that the second summand of the observed data influence function $\dot{\tau}(O;P)$ has mean zero given $(C, \sigma_C(X), S)$, as

$$\begin{aligned}
&S\,\mathbb{E}_P\left(\dot{\chi}(\boldsymbol{X}; P_{\boldsymbol{X}}^*) - \nu_C(\sigma_C(\boldsymbol{X}))\,|\, C, \sigma_C(X), S\right) \\
&= S\left\{\mathbb{E}_P\left(\dot{\chi}(\boldsymbol{X}; P_{\boldsymbol{X}}^*)\,|\, C, \sigma_C(\boldsymbol{X}), S = 1\right) - \nu_C(\sigma_C(\boldsymbol{X}))\right\} \\
&= 0,
\end{aligned}$$

by definition of $\nu_C(\sigma_C(\boldsymbol{X}))$. It follows that the two summands are uncorrelated, and

$$\begin{aligned}
\text{Var}_P(\dot{\tau}(O;P)) = {}& \text{Var}_P(\nu_C(\sigma_C(\boldsymbol{X}))) \\
&+ \text{Var}_P\left(\frac{S}{\eta(C, \sigma_C(\boldsymbol{X}))} \left\{ \dot{\chi}(\boldsymbol{X}; P_{\boldsymbol{X}}^*) - \nu_C(\sigma_C(\boldsymbol{X})) \right\} \right).
\end{aligned}$$

By the law of total variance, and given that the second summand has mean zero given

$(C, \sigma_C(X), S)$,

$$
\begin{aligned}
&\mathrm{Var}_P \left(\frac{S}{\eta(C, \sigma_C(\boldsymbol{X}))} \{\dot{\chi}(\boldsymbol{X}; P^*_{\boldsymbol{X}}) - \nu_C(\sigma_C(\boldsymbol{X}))\} \right) \\
&= \mathbb{E}_P \left(\frac{S}{\eta(C, \sigma_C(\boldsymbol{X}))^2} \mathrm{Var}_P \left(\dot{\chi}(\boldsymbol{X}; P^*_{\boldsymbol{X}}) \mid C, \sigma_C(X), S = 1 \right) \right) \\
&= \mathbb{E}_P \left(\frac{1}{\eta(C, \sigma_C(\boldsymbol{X}))} \mathrm{Var}_P \left(\dot{\chi}(\boldsymbol{X}; P^*_{\boldsymbol{X}}) \mid C, \sigma_C(X), S = 1 \right) \right),
\end{aligned}
$$

where in the last equality we conditioned on $(C, \sigma_C(\boldsymbol{X}))$. Finally, as

$$
\mathrm{Var}_P \left(\dot{\chi}(\boldsymbol{X}; P^*_{\boldsymbol{X}}) \mid C, \sigma_C(X), S = 1 \right) = \mathrm{Var}_{P^*} \left(\dot{\chi}(\boldsymbol{X}; P^*_{\boldsymbol{X}}) \mid C, \sigma_C(X) \right),
$$

and

$$
\mathbb{E}_P \left(\dot{\chi}(\boldsymbol{X}; P^*_{\boldsymbol{X}}) \,|\, C, \sigma_C(\boldsymbol{X}), S = 1 \right) = \mathbb{E}_{P^*} \left(\dot{\chi}(\boldsymbol{X}; P^*_{\boldsymbol{X}}) \,|\, C, \sigma_C(\boldsymbol{X}) \right),
$$

by Assumption A.1, adding and subtracting

$$
\mathbb{E}_P \left(\mathrm{Var}_P \left(\dot{\chi}(\boldsymbol{X}; P^*_{\boldsymbol{X}}) \mid C, \sigma_C(X), S = 1 \right) \right)
$$

yields the result, again by the law of total variance. □

Proposition A.4. *Let P, $\widetilde{P}$ be arbitrary putative observed data distributions, induced by full data distributions P^*, $\widetilde{P}^*$, respectively, and assume that both P^* and $\widetilde{P}^*$ satisfy Assumptions A.1 and A.2. Then* $\mathrm{Bias}_\tau(\widetilde{P}; P) \coloneqq \mathbb{E}_P(\dot{\tau}(O; \widetilde{P})) + \tau(\widetilde{P}) - \tau(P)$ *is equal to* $\mathrm{Bias}_\chi(\widetilde{P}^*_{\boldsymbol{X}}; P^*_{\boldsymbol{X}}) + \mathbb{E}_P \left[\left(1 - \frac{\eta(C, \sigma_C(\boldsymbol{X}))}{\widetilde{\eta}(C, \sigma_C(\boldsymbol{X}))} \right) \{\widetilde{\nu}_C(\sigma_C(\boldsymbol{X})) - \check{\nu}_C(\sigma_C(\boldsymbol{X}))\} \right]$*, where* $\mathrm{Bias}_\chi(\widetilde{P}^*_{\boldsymbol{X}}; P^*_{\boldsymbol{X}})$ *is similarly defined to equal* $\mathbb{E}_{P^*}(\dot{\chi}(\boldsymbol{X}; \widetilde{P}^*_{\boldsymbol{X}})) + \chi(\widetilde{P}^*_{\boldsymbol{X}}) - \chi(P^*_{\boldsymbol{X}})$*,* $\widetilde{\nu}_C(\sigma_C(\boldsymbol{X})) \coloneqq \mathbb{E}_{\widetilde{P}}(\dot{\chi}(\boldsymbol{X}; \widetilde{P}^*_{\boldsymbol{X}}) \mid C, \sigma_C(\boldsymbol{X}), S = 1)$*, and* $\check{\nu}_C(\sigma_C(\boldsymbol{X})) \coloneqq \mathbb{E}_P(\dot{\chi}(\boldsymbol{X}; \widetilde{P}^*_{\boldsymbol{X}}) \mid C, \sigma_C(\boldsymbol{X}), S = 1)$.

Proof. Observe that

$$
\mathbb{E}_P \left(\check{\nu}_C(\sigma_C(\boldsymbol{X})) \right) = \mathbb{E}_{P^*}(\dot{\chi}(\boldsymbol{X}; \widetilde{P}^*_{\boldsymbol{X}})),
$$

by iterated expectations and Assumption A.1. Moreover,

$$
\begin{aligned}
&\mathbb{E}_P \left(\frac{S}{\widetilde{\eta}(C, \sigma_C(\boldsymbol{X}))} \left\{ \dot{\chi}(\boldsymbol{X}; \widetilde{P}^*_{\boldsymbol{X}}) - \widetilde{\nu}_C(\sigma_C(\boldsymbol{X})) \right\} \right) \\
&= \mathbb{E}_{P^*} \left(\frac{\eta(C, \sigma_C(\boldsymbol{X}))}{\widetilde{\eta}(C, \sigma_C(\boldsymbol{X}))} \left\{ \dot{\chi}(\boldsymbol{X}; \widetilde{P}^*_{\boldsymbol{X}}) - \widetilde{\nu}_C(\sigma_C(\boldsymbol{X})) \right\} \right), \\
&= \mathbb{E}_P \left(\frac{\eta(C, \sigma_C(\boldsymbol{X}))}{\widetilde{\eta}(C, \sigma_C(\boldsymbol{X}))} \{\check{\nu}_C(\sigma_C(\boldsymbol{X})) - \widetilde{\nu}_C(\sigma_C(\boldsymbol{X}))\} \right),
\end{aligned}
$$

where in the first equality we condition on $(C, \sigma_C(\boldsymbol{X}), \overline{\sigma}_C(\boldsymbol{X}))$, and in the second equality we condition on $(C, \sigma_C(\boldsymbol{X}))$ and use Assumption A.1. Hence,

$$
\begin{aligned}
&\mathbb{E}_P(\dot{\tau}(O; \widetilde{P})) \\
&= \mathbb{E}_P \left(\widetilde{\nu}_C(\sigma_C(\boldsymbol{X})) + \frac{S}{\widetilde{\eta}(C, \sigma_C(\boldsymbol{X}))} \left\{ \dot{\chi}(\boldsymbol{X}; \widetilde{P}^*_{\boldsymbol{X}}) - \widetilde{\nu}_C(\sigma_C(\boldsymbol{X})) \right\} \right), \\
&= \mathbb{E}_P \left(\check{\nu}_C(\sigma_C(\boldsymbol{X})) \right) + \mathbb{E}_P \left(\widetilde{\nu}_C(\sigma_C(\boldsymbol{X})) - \check{\nu}_C(\sigma_C(\boldsymbol{X})) \right) \\
&\quad + \mathbb{E}_P \left(\frac{\eta(C, \sigma_C(\boldsymbol{X}))}{\widetilde{\eta}(C, \sigma_C(\boldsymbol{X}))} \{\check{\nu}_C(\sigma_C(\boldsymbol{X})) - \widetilde{\nu}_C(\sigma_C(\boldsymbol{X}))\} \right), \\
&= \mathbb{E}_{P^*}(\dot{\chi}(\boldsymbol{X}; \widetilde{P}^*_{\boldsymbol{X}})) \\
&\quad + \mathbb{E}_P \left[\left(1 - \frac{\eta(C, \sigma_C(\boldsymbol{X}))}{\widetilde{\eta}(C, \sigma_C(\boldsymbol{X}))} \right) \{\widetilde{\nu}_C(\sigma_C(\boldsymbol{X})) - \check{\nu}_C(\sigma_C(\boldsymbol{X}))\} \right].
\end{aligned}
$$

The result is obtained by noticing that $\chi(P^*_{\boldsymbol{X}}) = \tau(P)$, $\chi(\widetilde{P}^*_{\boldsymbol{X}}) = \tau(\widetilde{P})$. □

Remark 1. By Proposition A.4, we expect the observed-data influence function-based estimator $\widehat{\tau}$ to at least partially inherit robustness properties of the complete data influence function-based estimator $\widehat{\chi}$. The second term in the bias expression can be rewritten

$$\mathbb{E}_{P^*}\left[\left(1 - \frac{\eta(C, \sigma_C(\boldsymbol{X}))}{\widetilde{\eta}(C, \sigma_C(\boldsymbol{X}))}\right)\left(\frac{\widetilde{\lambda}'_C(\overline{\sigma}_C(\boldsymbol{X}); \sigma_C(\boldsymbol{X}))}{\lambda'_C(\overline{\sigma}_C(\boldsymbol{X}); \sigma_C(\boldsymbol{X}))} - 1\right)\dot{\chi}(\boldsymbol{X}; \widetilde{P}^*_{\boldsymbol{X}})\right], \tag{2}$$

where λ'_k, $\widetilde{\lambda}'_k$ are conditional densities of $\overline{\sigma}_k(\boldsymbol{X})$ given $C = k, \sigma_k(\boldsymbol{X}), S = 1$ under P and $\widetilde{P}$, respectively, for any $k \neq \infty$. This term can be simplified in certain examples (e.g., in our running example), but generally also exhibits a double robust property: it is zero if either $\widetilde{\eta} = \eta$ or $\widetilde{\lambda} = \lambda$. Thus, when the double sampling probabilities $\eta(C, \sigma_C(\boldsymbol{X}))$ are known by design, this term is automatically zero, so that $\text{Bias}_\tau(\widetilde{P}; P) = \text{Bias}_\chi(\widetilde{P}^*_{\boldsymbol{X}}; P^*_{\boldsymbol{X}})$.

The variance formula of Proposition A.3 facilitates an analysis of the loss of efficiency that the estimator based on double sampling incurs, compared to a complete-data influence function-based estimator that uses $\boldsymbol{X}$ on the complete sample. On the other hand, in view of Theorem A.1, the bias formula in Proposition A.4 is essential for determining conditions under which $\widehat{\tau}$ will be asymptotically normal and with variance attaining the nonparametric efficiency bound.

To elaborate on the previous point, for many common functionals (e.g., the running example in Section 2), the complete data asymptotic bias term $\text{Bias}_\chi(\widehat{P}^*_{\boldsymbol{X}}; P^*_{\boldsymbol{X}})$ exhibits a "mixed bias" property (Robins et al., 2008; Rotnitzky et al., 2020), in that it involves the product of nuisance function estimation errors. Moreover, the additional term in the bias expression also has this property: it is zero if either $\widehat{\eta} = \eta$ or $\widehat{\lambda}_k = \lambda_k$ for all $k \neq \infty$. In particular, this additional term is guaranteed to be zero when the double sampling probabilities η are known by design. Thus, the proposed observed data influence function-based estimators inherit any robustness properties of their complete data counterparts when η is known, and otherwise will have a slightly more elaborate multiple robustness structure due to the additional term.

Another important consequence of the mixed bias property is that of "rate double robustness" (Rotnitzky et al., 2020). Specifically, in the complete data setting, if $\text{Bias}_\chi(\widehat{P}^*_{\boldsymbol{X}}; P^*_{\boldsymbol{X}})$ depends on the product of $L_2(P)$-norm errors for estimating a pair of nuisance functions, and if this product converges to zero at rate $n^{-1/2}$, then the complete data influence function-based estimator will achieve $n^{-1/2}$ rate inference. The benefit is that this allows for more flexible estimation (e.g., errors converging faster than $n^{-1/4}$) of each of the nuisance functions, and the required convergence rates can be achieved by state-of-the-art machine learning models under smoothness or sparsity conditions, for example. In our case, we require the additional property that either η is known, or else $\|\widehat{\eta} - \eta\| \|\widehat{\lambda}'/\lambda' - 1\| = o_P(n^{-1/2})$, where λ' is the density corresponding to λ.

Finally, by the asymptotic normality result of Theorem A.1, a simple asymptotic variance estimator can obtained from the empirical variance of the estimated influence functions. The variance estimate for general functional $\tau(P)$ is $n \cdot \widehat{\text{Var}}(\widehat{\tau}) = \frac{1}{n}\sum_{k=1}^K \sum_{i \in I_k} (\dot{\tau}(O_i; \widehat{P}_k))^2$. Further, a Wald-type confidence interval is given by $\widehat{\tau} \pm z_{1-\alpha/2}\sqrt{\widehat{\text{Var}}(\widehat{\tau})}$, with z_α denoting the α-quantile of the standard normal distribution.

B Semiparametric results under Assumption 1

The observed data density is given by

$$p_o(O) = p_o(\boldsymbol{L}, A, R) p_o(Y \mid \boldsymbol{L}, A, R=1)^R p_o(S \mid \boldsymbol{L}, A, R=0)^{1-R} p_o(Y \mid \boldsymbol{L}, A, S=1)^S,$$

by Assumption 2 and the assertion that $R=1$ implies $S=0$, i.e., $S \equiv S(1-R)$. Under the MAR assumption (i.e., Assumption 1, $R \perp\!\!\!\perp Y \mid \boldsymbol{L}, A$), and Assumption 2, $S \perp\!\!\!\perp Y \mid \boldsymbol{L}, A, R=0$, we can conclude that

$$(R, S) \perp\!\!\!\perp Y \mid \boldsymbol{L}, A. \tag{3}$$

As a result, the conditional densities $p_o(Y \mid \boldsymbol{L}, A, R=1)$ and $p_o(Y \mid \boldsymbol{L}, A, S=1)$ are equal to $p_c(Y \mid \boldsymbol{L}, A) = p_o(Y \mid \boldsymbol{L}, A, R+S=1)$. Thus, the final observed data distribution P_o belongs to the semiparametric model induced by MAR if and only if its density can factorized according to

$$p_o(O) = p_o(\boldsymbol{L}, A, R) p_o(S \mid \boldsymbol{L}, A, R=0)^{1-R} p_o(Y \mid \boldsymbol{L}, A, R+S=1)^{R+S}.$$

By Lemma 24 of Rotnitzky and Smucler (2020), the tangent space of the semiparametric model at P_o is

$$\Lambda_{P_o} = \Lambda_{\boldsymbol{L}} \oplus \Lambda_{A \mid \boldsymbol{L}} \oplus \Lambda_{R \mid \boldsymbol{L}, A} \oplus (1-R)\Lambda_{S \mid \boldsymbol{L}, A, R} \oplus (R+S)\Lambda_{Y \mid \boldsymbol{L}, A, R+S},$$

where for any random vectors $\boldsymbol{W}, \boldsymbol{V}$, $\Lambda_{\boldsymbol{W} \mid \boldsymbol{V}} = \{a(\boldsymbol{W}, \boldsymbol{V}) \in L_2(P_o) : \mathbb{E}_{P_o}(a(\boldsymbol{W}, \boldsymbol{V}) \mid \boldsymbol{V}) = 0\}$. Now, under the MAR semiparametric model, $\tau_a^*(P_o) = \mathbb{E}_{P_o}(\mu_{a,\mathrm{MAR}}(\boldsymbol{L})) = \tau_a(P_o)$, where $\mu_{a,\mathrm{MAR}}(\boldsymbol{L}) = \mathbb{E}_{P_o}(Y \mid \boldsymbol{L}, A=a, R+S=1)$, as

$$\begin{aligned}
\mu_a(\boldsymbol{L}) &= \mu_{a,R}(\boldsymbol{L})\gamma_a(\boldsymbol{L}) + \mu_{a,S}(\boldsymbol{L})(1-\gamma_a(\boldsymbol{L})) \\
&= \mu_{a,\mathrm{MAR}}(\boldsymbol{L})\gamma_a(\boldsymbol{L}) + \mu_{a,\mathrm{MAR}}(\boldsymbol{L})(1-\gamma_a(\boldsymbol{L})) \\
&= \mu_{a,\mathrm{MAR}}(\boldsymbol{L}),
\end{aligned}$$

by (3). Moreover, the nonparametric influence function of $\tau_a^*(P_o)$ is simply

$$\dot{\tau}_{a,\mathrm{MAR}}(O; P_o) = \mu_{a,\mathrm{MAR}}(\boldsymbol{L}) - \tau_a^*(P_o) + \frac{T}{P_o[T=1 \mid \boldsymbol{L}]}(Y - \mu_{a,\mathrm{MAR}}(\boldsymbol{L})),$$

where $T = (R+S)\mathbb{1}(A=a)$ — viewing T as a modified treatment indicator, the influence function must be of the same form as that for the usual counterfactual mean functional (Hahn, 1998). The modified treatment probability can be expanded to

$$\begin{aligned}
P_o[T=1 \mid \boldsymbol{L}] &= P_o[A=a \mid \boldsymbol{L}] P_o[R=1 \vee S=1 \mid \boldsymbol{L}, A=a] \\
&= \pi_a(\boldsymbol{L})\{\gamma_a(\boldsymbol{L}) + (1-\gamma_a(\boldsymbol{L}))\eta_{a,0}(\boldsymbol{L})\}.
\end{aligned}$$

Finally, we notice that $\dot{\tau}_{a,\mathrm{MAR}}(O; P_o)$ must be the efficient influence function under P_o, as it belongs to Λ_{P_o}. To see this, note that $\mu_{a,\mathrm{MAR}}(\boldsymbol{L}) - \tau_a^*(P_o)$ is a mean-zero function of $\boldsymbol{L}$, so belongs to $\Lambda_{\boldsymbol{L}}$, and $\frac{T}{P_o[T=1 \mid \boldsymbol{L}]}(Y - \mu_{a,\mathrm{MAR}}(\boldsymbol{L}))$ has mean zero given $\boldsymbol{L}, A, R+S$, so belongs to $(R+S)\Lambda_{Y \mid \boldsymbol{L}, A, R+S}$.

Suppose now that we are not willing to assume that P_o belongs to the semiparametric model induced by MAR. Under mild assumptions (e.g., similar to Theorem A.1), an influence function-based estimator using $\dot{\tau}_{a,\mathrm{MAR}}(O; P_o)$ will be consistent for $\tau_a^*(P_o)$. However, we will

generally incur some bias because $\tau_a^*(P_o)$ is now not guaranteed to equal $\tau_a(P_o)$. Specifically, observe that

$$\begin{aligned}
&\mu_{a,\mathrm{MAR}}(\boldsymbol{L})\\
&= \mathbb{E}_{P_o}(Y \mid \boldsymbol{L}, A = a, R + S = 1)\\
&= \mathbb{E}_{P_o}\left[\mathbb{E}_{P_o}(Y \mid \boldsymbol{L}, A = a, R, S, R + S = 1) \mid \boldsymbol{L}, A = a, R + S = 1\right]\\
&= \mu_{a,R}(\boldsymbol{L}) P_o[R = 1 \mid \boldsymbol{L}, A = a, R + S = 1] + \mu_{a,S}(\boldsymbol{L}) P_o[S = 1 \mid \boldsymbol{L}, A = a, R + S = 1]\\
&= \frac{\mu_{a,R}(\boldsymbol{L})\gamma_a(\boldsymbol{L}) + \mu_{a,S}(1 - \gamma_a(\boldsymbol{L}))\eta_{a,0}(\boldsymbol{L})}{\gamma_a(\boldsymbol{L}) + (1 - \gamma_a(\boldsymbol{L}))\eta_{a,0}(\boldsymbol{L})}.
\end{aligned}$$

Thus, the bias of the outcome model is given by

$$\begin{aligned}
&\mu_{a,\mathrm{MAR}}(\boldsymbol{L}) - \mu_a(\boldsymbol{L})\\
&= \frac{\mu_{a,R}(\boldsymbol{L})\gamma_a(\boldsymbol{L}) + \mu_{a,S}(1 - \gamma_a(\boldsymbol{L}))\eta_{a,0}(\boldsymbol{L})}{\gamma_a(\boldsymbol{L}) + (1 - \gamma_a(\boldsymbol{L}))\eta_{a,0}(\boldsymbol{L})} - \mu_{a,R}(\boldsymbol{L})\gamma_a(\boldsymbol{L}) - \mu_{a,S}(1 - \gamma_a(\boldsymbol{L}))\\
&= \gamma_a(\boldsymbol{L}) \frac{(1 - \gamma_a(\boldsymbol{L}))(1 - \eta_{a,0}(\boldsymbol{L}))}{1 - (1 - \gamma_a(\boldsymbol{L}))(1 - \eta_{a,0}(\boldsymbol{L}))} (\mu_{a,R}(\boldsymbol{L}) - \mu_{a,S}(\boldsymbol{L})),
\end{aligned}$$

and the overall bias $\tau_a^*(P_o) - \tau_a(P_o)$ is the expectation of this quantity.

C Additional Details for Case Study Analysis

	**Surgery patients**
Number	13514
Sleeve Gastrectomy [surgery type] (%)	4659 (34.5)
Health care site (%)	
Washington	606 (4.5)
Northern California	3484 (25.8)
Southern California	9424 (69.7)
Year of surgery (%)	
2005	331 (2.4)
2006	824 (6.1)
2007	1220 (9.0)
2008	1424 (10.5)
2009	1711 (12.7)
2010	2237 (16.6)
2011	3281 (24.3)
2012	2486 (18.4)
Years of age at surgery (mean (SD))	46.29 (11.04)
Age categories (%)	
1: Age < 45	5939 (43.9)
2: $45 \leq$ Age < 65	7019 (51.9)
3: Age ≥ 65	556 (4.1)
Male [gender] (%)	2233 (16.5)
Race/ethnicity (%)	

Black	2471 (18.3)
Hispanic	4167 (30.8)
Unknown/Other	469 (3.5)
White	6407 (47.4)
Days of health care use 7-12 months pre-surgery (mean (SD))	9.24 (7.36)
Insulin use (%)	1651 (12.2)
Charlson/Elixhauser comorbidity score (%)	
-1	2667 (19.7)
0	5147 (38.1)
1	3249 (24.0)
2	2451 (18.1)
Hypertension diagnosis (%)	8063 (59.7)
ACE inhibitor use (%)	3437 (25.4)
ARB use (%)	1199 (8.9)
Other antihypertensive medication (%)	5801 (42.9)
Insurance type (%)	
Commercial	12096 (89.5)
Medicaid	488 (3.6)
Medicare	930 (6.9)
Diabetes status (%)	4956 (36.7)
Days hospitalized in year pre-surgery (mean (SD))	0.34 (1.81)
Dyslipidemia diagnosis (%)	6474 (47.9)
Statin use (%)	3667 (27.1)
Other lipid-lowering medication (%)	422 (3.1)
Smoking status (%)	
Ever, Self-Report	4684 (34.7)
Never, Self-Report	7468 (55.3)
No Self-Report	1362 (10.1)
Coronary artery disease (%)	355 (2.6)
Mental health diagnoses (%)	
Mild-Moderate Anxiety/Depression	5822 (43.1)
None	6096 (45.1)
Other	1596 (11.8)
Retinopathy (%)	615 (4.6)
Neuropathy (%)	945 (7.0)
Baseline BMI (mean (SD))	44.55 (7.12)

Table S1: Baseline characteristics of patients who underwent bariatric surgery, 2005–2015

Table S2: Numerical results for data application in Section 6, MAR setting. Results compared across second-stage sample sizes ($\sum_{i=1}^{n} S_i$), against full data benchmark (-6.83 [S.E. = 0.118]).

$\sum_{i=1}^{n} S_i$	Strategy #	Value	% "Bias"	Estimated S.E.
500	1	-6.98	2.08	0.133
	2	-6.93	1.48	0.169
	3	-7.00	2.42	0.131
	5	-7.00	2.42	0.166
1,000	1	-6.98	2.08	0.133
	2	-7.07	3.50	0.158
	3	-6.97	1.97	0.129
	5	-6.97	1.97	1.65
1,500	1	-6.98	2.08	0.133
	2	-6.99	2.28	0.142
	3	-6.97	2.01	0.127
	5	-6.97	2.01	0.141

Estimated S.E., estimated standard error; % "Bias", (value $-$ full data benchmark)/(full data benchmark) $\times$ 100;

Table S3: Numerical results for data application in Section 6, MNAR setting. Results compared across second-stage sample sizes ($\sum_{i=1}^n S_i$), against full data benchmark (-6.83 [S.E. $=$ 0.118]).

$\sum_{i=1}^n S_i$	Strategy #	Value	% "Bias"	Estimated S.E.
500	1	-8.25	20.71	0.121
	2	-6.91	1.07	0.178
	3	-7.93	15.99	0.122
	5	-6.91	1.07	0.178
1,000	1	-8.25	20.71	0.121
	2	-6.86	0.42	0.153
	3	-7.84	14.74	0.125
	5	-6.86	0.42	0.150
1,500	1	-8.25	20.71	0.121
	2	-6.89	0.83	0.139
	3	-7.59	11.13	0.124
	5	-6.89	0.83	0.141

Estimated S.E., estimated standard error; % "Bias", (value $-$ full data benchmark)/(full data benchmark) $\times$ 100;

Bickel, P., C. Klaassen, Y. Ritov, and J. Wellner (1993). *Efficient and Adaptive Estimation for Semiparametric Models*. Johns Hopkins University Press Baltimore.

Chernozhukov, V., D. Chetverikov, M. Demirer, E. Duflo, C. Hansen, W. Newey, and J. Robins (2018). Double/debiased machine learning for treatment and structural parameters. *The Econometrics Journal 21*(1), C1–C68.

Hahn, J. (1998). On the role of the propensity score in efficient semiparametric estimation of average treatment effects. *Econometrica 66*(2), 315–331.

Kennedy, E. H. (2020). Efficient nonparametric causal inference with missing exposure information. *The International Journal of Biostatistics 16*(1), 20190087.

Kennedy, E. H., S. Balakrishnan, and M. G'Sell (2020). Sharp instruments for classifying compliers and generalizing causal effects. *The Annals of Statistics 48*(4), 2008–2030.

Robins, J., L. Li, E. Tchetgen, and A. van der Vaart (2008). Higher order influence functions and minimax estimation of nonlinear functionals. In *Probability and Statistics: Essays in Honor of David A. Freedman*, pp. 335–421. Institute of Mathematical Statistics.

Robins, J. M., A. Rotnitzky, and L. P. Zhao (1994). Estimation of regression coefficients when some regressors are not always observed. *JASA 89*(427), 846–866.

Rotnitzky, A. and E. Smucler (2020). Efficient adjustment sets for population average causal treatment effect estimation in graphical models. *Journal of Machine Learning Research 21*, 1–86.

Rotnitzky, A., E. Smucler, and J. Robins (2020). Characterization of parameters with a mixed bias property. *Biometrika 106*(4), 875–888.

Tsiatis, A. (2007). *Semiparametric Theory and Missing Data*. Springer Sc. & Bus. Media.

Van der Vaart, A. W. (2000). *Asymptotic Statistics*, Volume 3. Cambridge University Press.
